# Authority endorsements backfire and social norms fail to increase vaccination intent in post-COVID Kazakhstan

**DOI:** 10.1016/j.ssmph.2026.101935

**Published:** 2026-06-20

**Authors:** David Karpa, Dinara Pisareva, Bermond Scoggins, Nikita Durnev, Michael Rochlitz

**Affiliations:** a Technical University of Munich, Germany; b Nazarbayev University, Astana, Kazakhstan; c Australian National University, Canberra, Australia; d Columbia University, New York, NY, United States; e University of Oxford, Oxford, United Kingdom

**Keywords:** Vaccination communication, Social learning theory, Prestige bias, Social norms, Kazakhstan, Survey experiment

## Abstract

Prestige bias and social norms messaging are among the most widely recommended vaccination communication strategies, yet they have been validated almost exclusively in high-trust, Western settings. Whether these strategies transfer to contexts where institutional trust is structurally low remains largely untested. We address this gap using a factorial survey experiment (n=1420 parents) in Kazakhstan – a post-Soviet setting where institutional trust is characterized by Soviet legacies and post-pandemic coercion – that varied messenger endorsement (Grand Mufti, President, Chief Sanitary Doctor, control) and social norm framing (Muslim, national, local, control), supplemented by qualitative analysis of 829 open-ended responses. All three endorsements reduced vaccination intent by 6–7 percentage points relative to the 76% control baseline, while norm messaging showed null effects overall. Non-Muslims drove the backfire (11–12 percentage point decline); Muslims were insulated. Urban residents responded positively to norms; rural residents did not. Qualitative analysis traced hesitancy to pragmatic safety and efficacy concerns – only two respondents cited religion – indicating the endorsement strategy targeted barriers that did not drive hesitancy in this population. These findings identify trust-dependent boundary conditions for prestige bias theory in health communication: when institutional trust is low and recent coercion has primed reactance, endorsements from prestigious figures trigger resistance rather than deference. The contrast with positive endorsement effects during the pandemic in the same country suggests that effectiveness depends on timing and whether attitudes are still forming. Where institutions have lost credibility, leveraging institutional authority is not just ineffective but counterproductive.

## Highlights

• Authority endorsements reduced vaccination intent by 6–7 pp.
• Backfire uniform across religious, political, medical endorsers.
• Social norm messaging null overall but positive in urban areas.
• Results identify trust-dependent boundary conditions for prestige bias.

## 1 Introduction

Two strategies grounded in social learning theory are prominent in vaccination communication research: prestige bias, which leverages endorsements from high-status figures, and social norms messaging, which highlights majority behavior within reference groups. Both have been validated almost exclusively in Western, industrialized contexts with comparatively high institutional trust (Prall, 2024, Prall et al., 2024). Whether these strategies work in settings where audiences distrust the very institutions being leveraged remains largely untested, yet precisely this question determines whether evidence-based messaging generalizes beyond the populations in which it was developed.

Both mechanisms rest on implicit trust assumptions. Prestige bias assumes that audiences grant epistemic authority to endorsers: people defer to high-status figures because they trust that status correlates with competence or reliable information (Jiménez & Mesoudi, 2019). Social norms messaging assumes that stated norms are credible: people adjust behavior toward perceived majority behavior because they trust the information source (Rendell et al., 2011). When institutional trust is high, these assumptions hold. When it is low, endorsements may highlight distrusted institutions rather than leveraging their authority, and stated norms may lack credibility (Palmer & Gorman, 2025). The broader vaccination communication literature reinforces this concern: fear-based messaging is only conditionally effective (Witte & Allen, 2000), social norms interventions may not survive publication-bias correction (Papakonstantinou et al., 2025), and the messenger often matters as much as the message content (Greyling et al., 2016).

Kazakhstan provides a critical test case for these trust-dependent mechanisms. Cross-national data indicate that Russia and several former Soviet republics, including Kazakhstan, exhibit relatively low trust in scientists compared to global averages (Cologna et al., 2025, Wellcome Trust, 2021). Vaccine confidence has been declining in multiple countries, including the post-Soviet states Azerbaijan and Georgia (de Figueiredo et al., 2020), and Kazakhstan ranks lowest in vaccine confidence among six Eastern European and Central Asian countries (35.5%) (Eagan et al., 2025). Approximately 35% of Kazakhstani parents exhibit hesitancy toward routine childhood immunization (Akhmetzhanova et al., 2020), parental refusal rates increased 2.62-fold between 2013 and 2022 (Abenova et al., 2024), and a measles resurgence has been characterized as an urgent public health crisis (Akilbekova et al., 2024, UNICEF, 2025). This combination of low institutional trust and rising hesitancy makes Kazakhstan an ideal setting to test whether WEIRD-validated communication strategies transfer to non-WEIRD, low-trust populations.

We conduct a factorial survey experiment with 1420 parents in Kazakhstan, testing messenger endorsements (Grand Mufti, President, Chief Sanitary Doctor) crossed with social norm framing (Muslim, national, local reference groups). Our design builds on Hicken et al. (2024), who found that religious endorsement reduced COVID-19 vaccine hesitancy in Kazakhstan by 7.4 percentage points; we extend their work to routine childhood vaccination (MMR), add the Chief Sanitary Doctor to test domain-specific versus domain-general prestige, and introduce social conformity treatments. Complementing the experiment, we analyze open-ended responses from 829 respondents to examine how the broader population makes sense of vaccination.

Our findings speak to three literatures. First, we contribute to vaccination communication research by providing experimental evidence from a non-WEIRD, low-trust setting – a context where most existing evidence is observational or pandemic-specific (Diaz et al., 2025, Hicken et al., 2024, Moehring et al., 2023, Prall, 2024, Prall et al., 2024, Ruggeri et al., 2024). Second, we refine prestige bias theory in cultural evolution by showing that low institutional trust does not merely weaken prestige cues but reverses their direction, and that this reversal erases the domain-specificity distinction: the medical authority backfired as uniformly as the political and religious ones, suggesting that audiences stopped evaluating expertise and started resisting authority as such (Abu-Akel et al., 2021, Brand et al., 2021, Brujić, 2024, Heinrich et al., 2024, Jiménez and Mesoudi, 2019, Palmer and Gorman, 2025). Third, we contribute to research on social norms messaging by showing that telling parents “most people vaccinate” only shifted intentions where recipients could not check the claim against their own experience – in cities but not in rural communities where vaccination behavior is directly observable (Dempsey and Wood, 2025, Papakonstantinou et al., 2025, Rabb et al., 2022, Rendell et al., 2011, Vriens et al., 2023). Together, these results identify trust and verifiability as scope conditions for two foundational social learning strategies, conditions that existing theory has largely taken for granted.

Section 2 reviews the literature and derives hypotheses, Section 3 describes the data and methods, Section 4 presents results, and Section 5 discusses implications.

## 2 Literature review

Social learning theory provides a useful framework for understanding vaccination communication: when direct experience with vaccine-preventable diseases is rare, people acquire information through observation, relying on social learning strategies such as prestige bias (learning from high-status individuals) and conformist bias (adopting behaviors perceived as common) (Rendell et al., 2011). We use this framework to structure our review and derive hypotheses.

### 2.1 Vaccine hesitancy in Kazakhstan

Vaccine confidence in Central Asia remains a significant public health challenge. Healthcare providers in Kazakhstan and Belarus exhibit the lowest confidence levels among six non-EU European countries surveyed across four regions, with only 77% agreeing that vaccines are important, safe, effective, and compatible with their beliefs (Claessens et al., 2025). These challenges reflect the broader legacy of post-Soviet healthcare transformation, which has eroded public trust in medical institutions across the region (Semenova et al., 2024): Soviet-era institutional exposure predicts lower vaccine confidence (Costa-Font et al., 2023), and qualitative evidence from Russia suggests vaccine hesitancy functions as an assertion of individual agency against perceived state overreach (Borozdina, 2025). Importantly, existing research suggests the drivers of hesitancy in Kazakhstan are pragmatic rather than ideological – parents who reject vaccination doubt vaccine effectiveness, distrust societal institutions, and perceive low disease threat – patterns documented for HPV vaccination (Aimagambetova et al., 2022, Babi et al., 2023) and childhood immunization (Yeskendir et al., 2023), while capability gaps, insufficient motivation, and limited opportunity are the primary barriers to immunization coverage (Gusmanov et al., 2023, Kassabekova et al., 2025).

Most directly relevant to our experimental design is the work of Hicken et al. (2024), who tested whether endorsement by religious leaders could reduce COVID-19 vaccine hesitancy across five countries. Results were largely null, but Kazakhstan was an important exception: religious endorsement decreased hesitancy by 7.4 percentage points, and individual religiosity did not moderate this effect. This raises a question: what mechanisms explain why messenger effects succeeded in Kazakhstan but failed elsewhere? We extend Hicken et al.’s work by testing whether their findings generalize from COVID-19 to routine childhood vaccination (MMR), adding the Chief Sanitary Doctor to compare domain-specific versus domain-general prestige, and introducing social conformity treatments to examine reference group effects.

In their synthesis of COVID-19 behavioral interventions, Ruggeri et al. (2024) identified the leadership effect and social consensus messaging as promising strategies, though effect sizes were modest and field replication was mixed – raising the question of whether these strategies perform differently outside the high-trust Western contexts in which most evidence was generated. Both fit within the social learning framework and motivate our two treatment arms. In a complementary LMIC context, Diaz et al. (2025) found that trust-based messaging from proximate health authorities was among the most effective approaches for HPV vaccine uptake in Colombia.

### 2.2 Prestige bias and messenger effects

Prestige bias leads people to learn from high-status individuals, an evolutionary shortcut that reduces the need to independently evaluate information sources (Henrich and Gil-White, 2001, Panchanathan, 2010). Empirical work documents its operation among adult learners facing novel problems (Atkisson et al., 2012) and in online social learning environments (Brand et al., 2020, Brand et al., 2021). This bias operates in both domain-specific and domain-general forms: people typically prefer domain experts when expertise is clearly relevant, but may defer to general prestige figures when domain expertise is unclear or when trust in domain experts is low (Jiménez & Mesoudi, 2019). Context shapes which type of prestige is most effective: expert endorsements increased willingness to share COVID-19 public health messages across six countries (Abu-Akel et al., 2021), while religious leaders played important roles in health communication during Ebola outbreaks in West Africa (Marshall, 2017). Prestige cues are also filtered through social identity: among Namibian pastoralists, in-group identity predicted vaccination learning preferences more strongly than domain expertise (Prall et al., 2024).

Given prestige bias theory and the positive effects observed by Hicken et al. (2024) in Kazakhstan, we hypothesize:

***H1**: Leadership-based messaging from prestigious figures will increase intent to vaccinate compared to non-attributed messaging.*

We expect these effects to vary across audiences: domain-specific prestige should outperform domain-general prestige where expertise is salient (Jiménez & Mesoudi, 2019), religious identity should amplify receptiveness to the Grand Mufti (Chu et al., 2021), and trust in government should condition the President’s effectiveness (Jennings et al., 2023, Nicholls et al., 2024). That said, Hicken et al. (2024) found no religiosity moderation in Kazakhstan, making H1b a direct replication test.

***H1a**: Domain-specific prestige (Chief Sanitary Doctor) messaging will be more persuasive than domain-general messaging.*

***H1b**: Religious prestige (Grand Mufti) messaging will be most persuasive among individuals who identify themselves as religious.*

***H1c**: Political prestige (President) messaging will be most persuasive among individuals with high trust in government.*

The positive findings on prestige bias largely come from contexts where baseline institutional trust can be assumed. When that trust is absent, endorsements from authorities can reverse belief updating, pushing audiences away from the endorsed position (Palmer & Gorman, 2025) – as suggested by the failure of WHO endorsement to mitigate country-of-origin bias in U.S. vaccine policy support (Heinrich et al., 2024) and the ineffectiveness of expert messaging in post-socialist Serbia (Brujić, 2024). Yet direct experimental evidence of endorsement backfire remains scarce, making the conditions under which prestige cues become counterproductive an open empirical question. Psychological reactance amplifies this dynamic: when messages are perceived as threatening freedom of choice, individuals resist rather than comply (Brehm, 1966, Brehm and Brehm, 1981), an effect linked to trait reactance, which is elevated among the vaccine-skeptical (Soveri et al., 2024). Whether endorsements ultimately help or harm thus depends on whether audiences grant the endorser credibility to make knowledge claims in the first place (Cummings, 2014, Kerr et al., 2021, Lalumera, 2018). In post-Soviet Kazakhstan, where trust in government institutions is structurally low (Costa-Font et al., 2023) and cross-national data indicate low trust in scientific authorities (Cologna et al., 2025), and where mandatory COVID-19 vaccination policies triggered lasting backlash against health authorities, these conditions cannot be assumed.

### 2.3 Conformist bias and social norms

Conformist bias drives people to adopt behaviors perceived as common within their reference groups (Rendell et al., 2011). Reference group selection proves critical: shared religious identity increased vaccination intentions among highly religious participants (Chu et al., 2021), while perceived co-partisan behavior strongly influenced Republicans’ vaccination attitudes (Rabb et al., 2022). A large-scale experiment (N = 484,239) demonstrated that normative information about others’ vaccination intentions can increase individuals’ own willingness to vaccinate, though effect sizes were modest (Moehring et al., 2023). Importantly, vaccine-hesitant individuals tend to misperceive social norms, systematically underestimating the prevalence of vaccination in their communities (Vriens et al., 2023), and a recent review confirms that perceived descriptive norms, and to a lesser extent injunctive norms, are associated with vaccine hesitancy across multiple studies (Dempsey & Wood, 2025).

Based on conformist bias theory, we expect that learning about high vaccination rates within one’s reference group will increase personal vaccination intent:

***H2**: Social conformity messaging highlighting reference group norms will increase intent to vaccinate compared to no-norms messaging.*

***H2a**: National conformity norms will be more persuasive than no conformity messaging.*

Because norm influence declines as the reference group grows larger and more diffuse (Rabb et al., 2022), local norms should carry more weight in regional areas where the community is smaller and vaccination behavior more directly observable:

***H2b**: Local community norms will be most persuasive among individuals from regional areas compared to major cities.*

Because shared religious identity can strengthen vaccination intentions through trust and shared values (Chu et al., 2021):

***H2c**: Religious community norms will be most persuasive among individuals who identify themselves as religious.*

Our heterogeneous treatment effect analyses (H1b, H1c, H2b, H2c) allow us to explore whether the trust- and reactance-related boundary conditions discussed above moderate these predicted effects.

## 3 Data and methods

### 3.1 Sample and recruitment

We conducted an online survey experiment with 3124 respondents in Kazakhstan in March 2025. The survey was carried out by the Astana-based polling company NAC Analytica. Participants were recruited through quota sampling on gender, age, and region. The survey was administered in both Kazakh and Russian, with respondents able to switch languages at any point. Participants received compensation on their phone balance for completing the survey.

Eligibility required being at least 18 years old and a resident of Kazakhstan. This study was approved by the Nazarbayev University Institutional Research Ethics Committee on September 20, 2024 (IREC #937/18092024) and by the Central University Research Ethics Committee (CUREC) of the University of Oxford (#662978). The study was pre-registered on AsPredicted (#191932) on September 29, 2024, prior to data collection.

### 3.2 Experimental design

We used a 4 × 4 between-subjects factorial design with two treatment dimensions: messenger attribution (4 levels) and social conformity framing (4 levels). All respondents were randomly assigned to one of 16 experimental conditions. Because our outcome measure focused on parental intentions to vaccinate children, our primary analysis is restricted to the 1420 respondents who indicated they had children under 18 years old; respondents without children under 18 answered a hypothetical version of the outcome question (see the outcome measure below) and serve as a robustness comparison.

The first treatment dimension varied who endorsed the MMR vaccine. All three messenger vignettes followed the same template:

“In order to combat measles and its severe side effects among children, [*Endorser*] endorsed the MMR vaccine to all citizens of Kazakhstan. [*Title*] publicly declared the vaccine to be safe and [*adjective*]”.

The Grand Mufti is the highest Sunni Muslim religious authority in Kazakhstan; the Chief Sanitary Doctor (*Главнюй санитарнюй врач*, a role roughly equivalent to Chief Medical Officer in the UK or Surgeon General in the US) oversees public health policy. The control condition showed no attribution statement. Each adjective reflects what the endorser would plausibly emphasize in practice, but this means we cannot fully isolate messenger from message content effects (see limitations). Table 1 reports the varying elements across conditions.

The second treatment dimension varied which reference group was cited as having vaccinated their children:

“4 out of 5 [*reference group*] have already vaccinated their children with the MMR vaccine”.

The control condition showed no conformity statement. The “4 out of 5” statistic was hypothetical; respondents who found it implausible may have discounted the treatment.

**Table 1:** Experimental treatment conditions.

Dimension	Condition	Varying element	Adjective
Messenger	Grand Mufti	Grand Mufti of Kazakhstan Nauryzbai Otpenov	halal
	President	President Kassym-Jomart Tokayev	patriotic
	CSD	Chief Sanitary Doctor Sarkhat Beisenova	effective
	Control	*No attribution statement*	

Conformity	Religious	Muslims in Kazakhstan	–
	National	Citizens in Kazakhstan	–
	Local	Residents in your area	–
	Control	*No conformity statement*	

### 3.3 Outcome measure

The primary outcome was vaccination intention, measured by the question: ‘Do you plan to vaccinate your children against measles, mumps, and rubella, or have they already been vaccinated? Hypothetically, if you did not have children, would you vaccinate them?’ with response options ‘yes’ and ‘no’. This measure combines future vaccination intent with past behavior (children already vaccinated), which means that treatment effects are estimated among a mix of respondents for whom vaccination is a prospective decision and those reporting an already-completed action. To the extent that parents whose children are already vaccinated are less susceptible to messaging effects, our estimates may understate effects among the truly undecided.

### 3.4 Pre-treatment covariates

The survey collected extensive demographic and attitudinal measures prior to treatment exposure. Demographic variables included age, gender, region of residence, ethnicity (Kazakh, Russian, other), religious affiliation (Islam, Orthodox, other, non-religious), frequency of religious practice, education level, employment status, number of children, financial situation, and settlement type (major city, regional center, village). Language variables captured which languages respondents know well, which they use at home, at work/school, and for news consumption. Media consumption patterns and sources for political and economic news were also measured.

Attitudinal variables included generalized social trust (‘To what extent can most people be trusted?’), trust in the government of Kazakhstan, and opinions toward Russia, China, and the United States. These measures allow us to test hypothesized heterogeneous treatment effects (religiosity for H1b and H2c, government trust for H1c, urban/rural residence for H2b). Table 2 reports descriptive statistics for the full sample and parent subsample.

### 3.5 Open-ended qualitative measure

All survey respondents were asked a voluntary open-ended question about their general vaccination experiences: ‘Could you describe your experience with vaccinations and your reasons for supporting or not supporting vaccination of yourself and/or family members?’. Of the 3124 respondents, 829 (27%) provided responses, which we analyze qualitatively in Section 4.2.

The qualitative data address our second research question: how does the broader population in Kazakhstan make sense of vaccination? Because the open-ended question asked about general vaccination experiences rather than MMR-specific intentions, and was posed to all respondents rather than only parents, the qualitative findings provide context about vaccination attitudes in Kazakhstan that complements but is distinct from the experimental analysis.

### 3.6 Analytical strategy

In analyzing the factorial experiment, we estimate main effects (i.e. the effect of one row while averaging over the other) and interaction effects using linear probability models with heteroskedasticity-robust (HC2) standard errors. To aid interpretation of the results, we report predicted probabilities of vaccination intent, as well as the contrasts between the treatment and the control conditions. For our analysis of heterogeneous main effects, we interact the endorsement and norm treatment variables with moderator variables motivated by our hypotheses (religious identity for H1b and H2c, government trust for H1c, urban/rural residence for H2b). Given the exploratory nature of interaction analyses with multiple comparisons, we interpret these results with appropriate caution and emphasize point estimates alongside confidence intervals rather than relying solely on statistical significance.

With approximately 89 respondents per cell in the 4 × 4 design, the experiment is adequately powered to detect main effects of 6–7 percentage points but has limited power for detecting heterogeneous treatment effects in subgroup analyses. We interpret subgroup analyses with appropriate caution and emphasize patterns across multiple comparisons rather than individual significant contrasts.

For the qualitative analysis, we used inductive thematic coding in Atlas.ti. Coding proceeded in two stages: initial open coding across all 829 responses (650 in Russian, 179 in Kazakh), followed by organization into higher-order categories. All coding was conducted by a single researcher. Code definitions, merging decisions, and category assignments were documented at each stage. A second team member reviewed the final codebook against a random sample of 80 responses (approximately 10 percent) to verify consistency and flag interpretive disagreements. We acknowledge that single-coder analysis is a limitation, though we note that the primary qualitative findings (the predominance of pragmatic safety and institutional trust concerns) are descriptive patterns unlikely to be substantially affected by coder variation.

## 4 Results

### 4.1 Experimental results

#### 4.1.1 Main effects

Contrary to H1, leadership-based messaging significantly decreased vaccination intent relative to the control condition. Among the 1420 respondents with children under 18, the control group exhibited a baseline vaccination intent of 76.0%. All three messenger conditions produced statistically significant or marginally significant negative effects: President Tokayev (β=−0.074, p<.05), the Grand Mufti of Kazakhstan (β=−0.066, p<.05), and the Chief Sanitary Doctor (β=−0.063, p<.10, where marginal significance indicates p-values between .05 and .10). H1 is not supported.

We also find no support for H1a: the Chief Sanitary Doctor condition, representing domain-specific medical expertise, did not outperform the domain-general messengers. The similar magnitude across all three endorsers is notable and discussed further in Section 5.

**Fig. 1:**
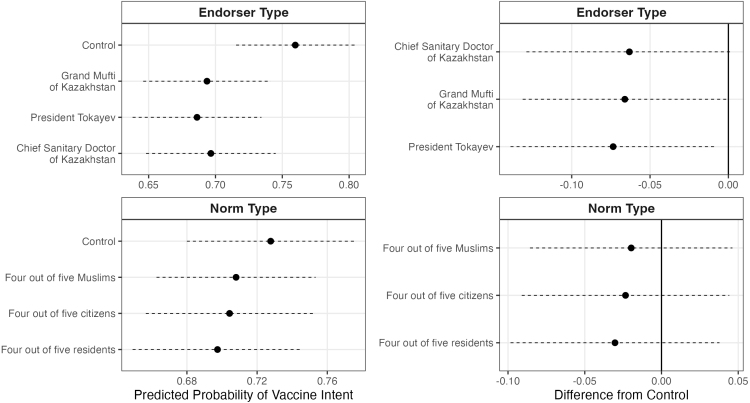
Main treatment effects on vaccination intent among parents (n = 1420). Left panels show predicted probabilities of vaccination intent for each treatment condition with 95% confidence intervals; right panels show differences from the control condition (baseline intent = 76.0%). Top row: endorser type effects – Grand Mufti of Kazakhstan, President Tokayev, and Chief Sanitary Doctor each reduce intent by 6–7 percentage points relative to control. Bottom row: norm type effects – religious, national, and local community norms all produce small negative but non-significant effects. Estimates are from linear probability models with heteroskedasticity-robust standard errors.

We find no support for H2 or H2a. The main effects of norm type were uniformly negative but failed to reach statistical significance: local residents (β=−0.030, SE = 0.035), national citizens (β=−0.023, SE = 0.034), and religious community (β=−0.020, SE = 0.034). H2a specifically predicted that national conformity norms would increase intent relative to control, but the national norm condition produced a small negative (and non-significant) effect. These null findings suggest that simply citing majority vaccination behavior, regardless of whether the reference group is defined by religion, nationality, or geography, does not shift intentions in this population (Fig. 1; Table 3).

To assess whether the high baseline rate among already-vaccinated parents masks treatment effects among the truly undecided, we re-estimate the main models restricting the sample to parents whose children were not yet vaccinated at the time of the survey (n=529). In this subsample, baseline vaccination intent drops to approximately 39%, providing substantially more room for treatment effects to emerge. Yet none of the endorser or norm coefficients reach statistical significance, and all are close to zero (Table 4). This confirms that the null and negative findings in the full sample are not an artifact of ceiling effects among already-vaccinated parents. As a further check, we replicate the main models on the full survey sample including non-parents (N=3124); all treatment effects are near zero and insignificant (Supplementary Table 13), confirming that the backfire effect is specific to respondents facing actual vaccination decisions. Results are also robust to the inclusion of region fixed effects, with endorser treatment coefficients increasing slightly in magnitude and precision (Supplementary Table 14).

**Table 2:** Descriptive statistics.

	Unique	Missing Pct.	Mean	SD	Min	Median	Max
*Panel A: Full Sample (N = 3124)*							
Age	63	0	42.1	14.4	18.0	41.0	82.0
Female	2	0	0.5	0.5	0.0	1.0	1.0
Children	6	0	0.9	1.2	0.0	0.0	5.0
Employed	2	0	0.6	0.5	0.0	1.0	1.0
Russian at Home	2	0	0.5	0.5	0.0	0.0	1.0
Muslim	2	0	0.6	0.5	0.0	1.0	1.0
Religiosity	6	19	2.2	1.3	0.0	2.0	4.0
Higher Education	2	0	0.4	0.5	0.0	0.0	1.0

*Panel B: Parent Subsample (N = 1420)*							
Age	51	0	41.8	11.0	18.0	41.0	72.0
Female	2	0	0.6	0.5	0.0	1.0	1.0
Children	5	0	1.9	1.0	1.0	2.0	5.0
Employed	2	0	0.7	0.5	0.0	1.0	1.0
Russian at Home	2	0	0.5	0.5	0.0	0.0	1.0
Muslim	2	0	0.6	0.5	0.0	1.0	1.0
Religiosity	6	15	2.2	1.3	0.0	2.0	4.0
Higher Education	2	0	0.4	0.5	0.0	0.0	1.0

**Table 3:** Row-level treatment effects on vaccine intent.

	Endorser type	Norm type
Intercept (Control)	0.760***	0.728***
	(0.023)	(0.024)
Grand Mufti of Kazakhstan	−0.066*	
	(0.033)	
President Tokayev	−0.074*	
	(0.033)	
Chief Sanitary Doctor of Kazakhstan	−0.063+	
	(0.034)	
Four out of five Muslims		−0.020
		(0.034)
Four out of five citizens		−0.023
		(0.034)
Four out of five residents		−0.030
		(0.035)

Num. Obs.	1420	1420
R${}^{2}$	0.004	0.001
R${}^{2}$ Adj.	0.002	−0.002

*Notes:* + p < 0.1, * p < 0.05, ** p < 0.01, *** p < 0.001. Reference category is Control for both models.

**Table 4:** Treatment effects among not-yet-vaccinated parents.

	Endorser type	Norm type
Intercept (Control)	0.392***	0.356***
	(0.044)	(0.044)
Grand Mufti of Kazakhstan	−0.023	
	(0.061)	
President Tokayev	0.034	
	(0.061)	
Chief Sanitary Doctor	−0.036	
	(0.060)	
Four out of five Muslims		0.040
		(0.060)
Four out of five citizens		0.026
		(0.061)
Four out of five residents		0.049
		(0.062)

Num. Obs.	529	529
R${}^{2}$	0.003	0.001

*Notes:* + p < 0.1, * p < 0.05, ** p < 0.01, *** p < 0.001. Sample restricted to parents whose children were not yet vaccinated pre-treatment. Same specification as Table 3.

#### 4.1.2 Heterogeneous and interaction effects

The interaction plot and associated contrasts show no systematic interaction patterns between messenger and norm treatments. The negative main effects of messenger attribution appear to operate independently of the norm manipulation, with no evidence that particular messenger-norm combinations produce synergistic or countervailing effects (Supplementary Figures S1–S2).

The following subgroup analyses are motivated by our hypotheses: Muslim status moderating endorser effects (H1b), government trust moderating presidential endorsement effects (H1c), urban/rural residence moderating local norm effects (H2b), and Muslim status moderating religious norm effects (H2c). These interaction analyses were not individually specified in the pre-registration, which focused on the main experimental question and overall analytical approach. Results should be interpreted with the caveat that the study has limited statistical power for detecting interaction effects.

**Fig. 2:**
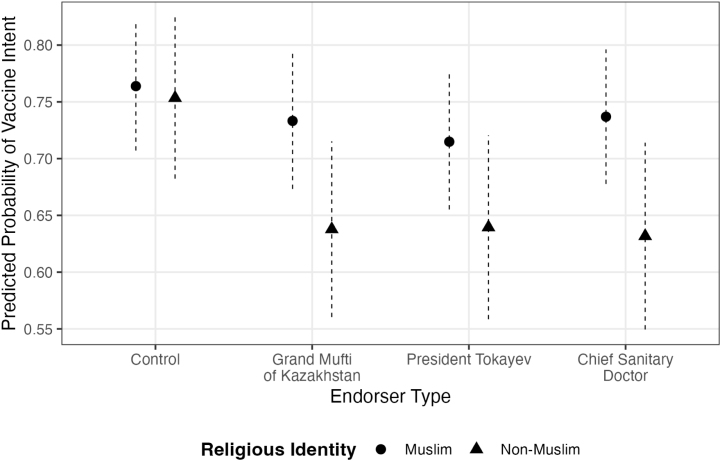
Heterogeneous endorser effects by Muslim status (n = 1420). Predicted probabilities of vaccination intent for Muslim (n≈852) and non-Muslim (n≈568) respondents across endorser conditions, with 95% confidence intervals. Muslims maintain relatively stable intent across conditions (≈71%–76%), while non-Muslims decline sharply from control (≈75%) to treatment conditions (≈63%–64%), a backfire effect of 11–12 percentage points. This pattern holds across all three endorsers, not only the Grand Mufti.

**Muslim and non-Muslim responses to religious endorsement (H1b).** H1b predicted that the Grand Mufti endorsement would be most persuasive among Muslims; this is not supported. The key finding is that non-Muslims drove the backfire effect, with intent declining by 11–12 percentage points across all three endorser conditions, while Muslims maintained stable intent (Fig. 2). This insulation pattern holds across all three endorsers, not just the Mufti. The contrast estimates are shown in Supplementary Figure S3. As an exploratory check, we re-estimated the interaction replacing Muslim status with ethnicity (ethnic Russian vs. ethnic Kazakh/Other; see Supplementary Table 9 and Supplementary Figures S4–S5). The ethnic split sharpens the divide observed for Muslim status: ethnic Russians decline by 15–19 percentage points across the three endorsers, while ethnic Kazakh/Other respondents show small and non-significant declines (1–3 pp). Within this exploratory specification, the Grand Mufti × Ethnic Russian interaction is statistically significant (β=−0.180, p<0.05), and the President × Ethnic Russian interaction is marginal (β=−0.144, p<0.1); the Chief Sanitary Doctor × Ethnic Russian interaction is not significant (β=−0.119, p>0.1). This leaves open the possibility of a Mufti-specific religious-outgroup channel for ethnic Russians, in addition to the broader ethnic-institutional pattern, but the comparison is not pre-registered and the study is underpowered to adjudicate between the two cleanly.

**High and low government trust responses to presidential endorsement (H1c).** H1c predicted that President Tokayev’s endorsement would be most persuasive among individuals with high government trust; this is not supported. High-trust individuals exhibited higher baseline intent (approximately 81% vs. 68%), but both groups declined similarly across all endorser conditions; government trust did not buffer the backfire effect. H1c is not supported (Supplementary Figures S6–S7).

**Fig. 3:**
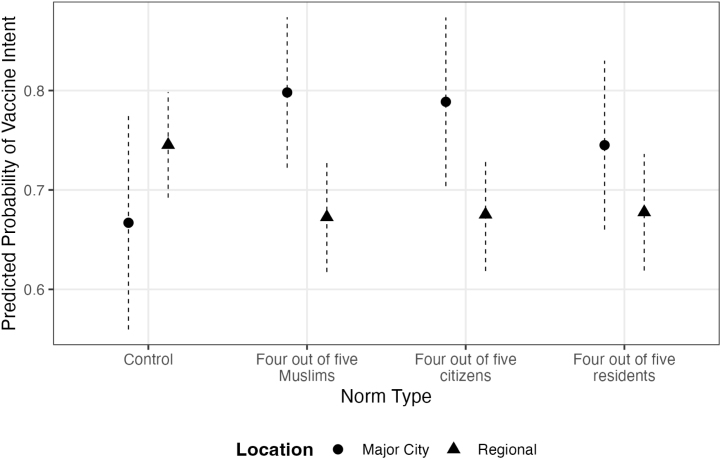
Heterogeneous norm effects by location type (n = 1420). Predicted probabilities of vaccination intent for major city (Almaty, Astana, Shymkent) and regional respondents across norm conditions, with 95% confidence intervals. Regional respondents maintain stable intent (≈68%–75%) across all conditions, while major city respondents show a striking increase from control (≈67%) to all three norm conditions (≈75%–80%). This urban–rural divergence is not specific to local norms: major city respondents respond similarly to religious, national, and local reference groups.

**Urban and rural responses to local community norms (H2b).** H2b predicted that local community norms would be most persuasive among regional residents; the opposite emerged. Major city respondents showed striking increases across all norm conditions, while regional respondents were unaffected (Fig. 3; Supplementary Figure S8). This pattern is not specific to local norms, though wide confidence intervals warrant caution about these exploratory interaction effects. H2b is not supported.

**Muslim status and religious community norms (H2c).** H2c predicted that religious community norms would be most persuasive among Muslims. The subgroup analysis shows no evidence of such moderation: Muslim status does not substantially moderate responses to social norm messaging, with both groups showing similar, largely null responses to all three norm treatments (Supplementary Figures S9–S10). H2c is not supported.

### 4.2 Qualitative analysis of open-ended responses

The qualitative analysis addresses our second research question: How does the broader population in Kazakhstan make sense of vaccination?

#### 4.2.1 Analytical approach and sample considerations

Among the 3124 survey respondents, 829 (27%) answered the optional open-ended question about their vaccination experiences and attitudes. We analyzed these responses using inductive thematic coding in Atlas.ti to identify dominant narratives. The coding proceeded in two stages: initial open coding across all 829 responses (650 in Russian, 179 in Kazakh), followed by organization into higher-order categories identifying two main dimensions: Attitudes (779 codes) and Practices (364 codes).

Respondents differed from non-respondents: younger/older age groups (26–35 and 65+), Russian speakers, Orthodox Christians, and those with lower government trust were overrepresented. Qualitative responses are also subject to multiple stages of self-selection and social desirability bias, which may shape both who responded and what they were willing to disclose.

#### 4.2.2 Attitudes toward vaccination

**Fig. 4:**
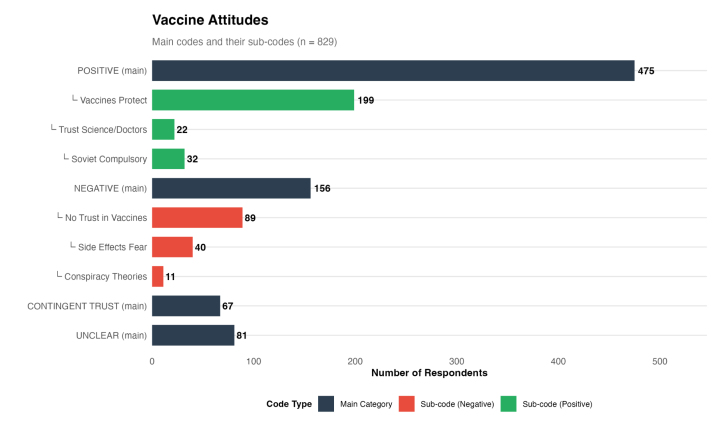
Distribution of vaccine attitude codes from qualitative analysis (n = 829). Respondents were asked an optional open-ended question about their vaccination experiences; 829 of 3124 survey participants (27%) provided responses. Codes were assigned inductively in Atlas.ti. The four main attitude categories (positive, negative, contingent trust, unclear) sum to 779 codes; some responses received multiple codes. Percentages reflect the share of attitude codes within each category.

We identified four main attitude categories (Fig. 4). Most respondents (61%) expressed positive attitudes, with the dominant narrative emphasizing that vaccines protect societies from disease (‘Thanks to vaccines, we survive’). A smaller subset endorsed Soviet-era compulsory vaccination practices, though this sentiment appeared exclusively among Russian speakers.

Around 20% expressed negative attitudes. Within this group, 57% expressed general distrust of vaccines while 26% cited specific fears about side effects. Vaccine skepticism was characterized primarily by pragmatic concerns such as doubts about efficacy and worries about adverse reactions rather than ideological opposition. Only two respondents mentioned religion as a reason for opposing vaccination. While social desirability may lead to underreporting, the near-absence of religious justifications suggests that religious objections are not a primary driver of hesitancy in Kazakhstan. Nine percent expressed contingent trust, with support depending on vaccine quality, doctor recommendations, or situational necessity. This category represents potentially persuadable individuals whose decisions depend on trust in regulatory processes.

#### 4.2.3 Vaccination practices

**Fig. 5:**
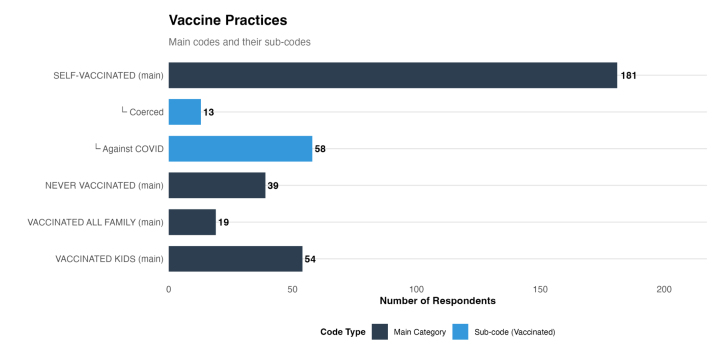
Distribution of vaccination practice codes from qualitative analysis (n = 829). Codes capture self-reported vaccination behaviors and experiences mentioned by respondents. Categories include personal vaccination history, COVID-19-specific experiences, coerced vaccination, and child vaccination. The 364 practice codes were assigned independently from the 779 attitude codes; a single response could receive both attitude and practice codes.

Of those who described practices (Fig. 5), 22% shared personal vaccination history (one-third mentioning COVID-19). Approximately 7% indicated they were coerced into vaccination, often citing COVID-19 requirements: ‘My last vaccination experience was only for COVID, which I didn’t fully agree with. I didn’t like the coercion from the government.’ Around 7% specifically mentioned vaccinating their children.

#### 4.2.4 Key demographic patterns

Analysis by demographics showed several notable patterns. Government trust showed the strongest association with vaccine attitudes: among those who fully trust the government, 75% expressed positive vaccination views versus only 40% among those with no trust. This 35-percentage-point gap underscores the centrality of government trust in shaping vaccination attitudes. Both religious and non-religious groups expressed predominantly positive attitudes, and combined with the near-absence of religious justifications for refusal, this suggests religiosity may not be a strong predictor of hesitancy. Men were slightly more skeptical (23% negative vs. 17% for women), while the 18–25 and 36–45 age groups showed highest skepticism. Regional variation was substantial, with Atyrau showing highest skepticism and Almaty highest support.

An unexpected finding was language switching: approximately 50% of respondents who indicated Kazakh as their home language nonetheless responded in Russian, potentially indicating that vaccination discourse is more developed in Russian (Supplementary Figure S11).

#### 4.2.5 Narrative discourses

**Fig. 6:**
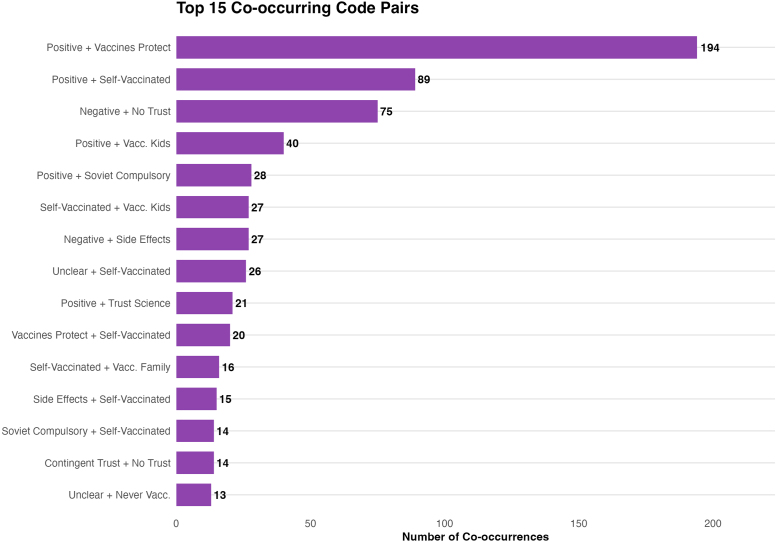
Top 15 co-occurring code pairs from qualitative analysis.

Analysis of code co-occurrences (Supplementary Figure S12; Fig. 6) identified four distinct discourses that structure vaccination attitudes in this population.

**Convinced Support** (Positive + Vaccines Protect + Self-Vaccinated + Trust in Science): A coherent pro-vaccine narrative expressed by people who believe vaccines work and act accordingly.

**Institutional Distrust** (Negative + No Trust + Contingent Trust): Broad skepticism directed at doctors, healthcare institutions, and vaccine quality rather than vaccines per se.

**Safety Concern** (Negative + Side Effects): Specific concerns about vaccine safety, representing pragmatic, health-focused objections.

**Reluctant Compliance** (Unclear + Self-Vaccinated + Side Effects): People who vaccinate despite reservations, suggesting external pressures may override personal hesitancy.

## 5 Discussion

Prestige bias theory assumes a trust baseline that does not hold universally. Our results identify boundary conditions for this theory: when institutional trust is structurally low and recent coercion has primed reactance, endorsements from prestigious figures reverse rather than persuade. All three authority endorsements reduced vaccination intent by 6–7 percentage points relative to the 76% control baseline, social norm messaging produced null effects overall, and heterogeneous effects revealed that Muslims were insulated from backlash, urban residents responded positively to norms while rural residents did not, and qualitative analysis traced hesitancy to pragmatic safety and efficacy concerns rather than religious objections or conspiracy theories. These findings suggest that the trust assumptions undergirding both prestige bias and conformist bias are not universal features of health communication but contingent on institutional context.

### 5.1 Reactance, trust, and the limits of prestige bias

When individuals perceive messages as threatening freedom of choice, they resist rather than comply (Brehm, 1966). Our context activated reactance on two levels. First, the explicit authority attribution signaled persuasive intent – controlling language is a known reactance trigger (Miller et al., 2007) – and phrases like “endorsed the MMR vaccine to all citizens” carry an imperative quality that gentler framings might avoid. Second, pandemic-era vaccination mandates had associated authority-based health messaging with coercion, as our qualitative data illustrate: “My last vaccination experience was only for COVID, which I didn’t fully agree with. I didn’t like the coercion from the government”. Pavey et al. (2024) show that moral obligation framing increases reactance specifically among the already hesitant, suggesting our endorsement treatments may have activated the very resistance they sought to overcome. The uniformity of backfire across all three endorsers – including the domain-specific expert (Chief Sanitary Doctor) – suggests respondents resisted the very act of authorities presuming to guide their health decisions, not any particular authority. Notably, the backfire effect was confined to parents; replicating the same models on the full sample including non-parents (N=3124) yields null effects across all treatment arms (Supplementary Table 13), suggesting that reactance is activated by having skin in the game – a real decision about one’s child – rather than by endorsement exposure alone.

The theoretical framework of Palmer and Gorman (2025) predicts that when audiences distrust information sources, belief updating can reverse direction: endorsements push beliefs *away* from the endorsed position. Our context exemplifies this dynamic. Soviet institutional exposure predicts vaccine distrust (Costa-Font et al., 2023), and qualitative evidence from Russia shows vaccine refusal functioning as an assertion of agency against perceived state overreach (Borozdina, 2025). The effectiveness of endorsements hinges on whether audiences recognize the endorser as a legitimate source of knowledge claims (Cummings, 2014, Kerr et al., 2021, Lalumera, 2018). In post-Soviet Kazakhstan, where trust in government institutions is structurally low (Costa-Font et al., 2023) and cross-national data indicate low trust in scientific authorities (Cologna et al., 2025), that recognition is withheld.

The backlash is not uniquely post-Soviet. DeMora et al. (2025) found that patriotism-framed COVID-19 vaccine messages backfired among U.S. Republicans, with the public health official – expected to be most persuasive – instead producing consistent backlash, a decrease of 5 to 8 points on a 100-point attitude scale. The parallel with our President-endorsed “safe and patriotic” treatment is striking: in both cases, framing vaccination as a civic duty activated resistance rather than compliance. Brujić (2024) documented similar dynamics in post-socialist Serbia, and Heinrich et al. (2024) found that WHO endorsement did not mitigate country-of-origin bias in U.S. vaccine policy preferences. Wherever audiences perceive endorsements as ideologically motivated or coercive, authority-based messaging can have reverse effects, regardless of whether the underlying trust deficit stems from post-Soviet legacies or partisan polarization.

Our results directly contradict Hicken et al. (2024), who found that religious endorsement reduced COVID-19 hesitancy by 7.4 percentage points in the same country. The most likely explanation is timing. Hicken et al. collected data during the pandemic when uncertainty was high, attitudes were forming, and guidance from authorities was sought. We collected data post-pandemic for a routine vaccine about which parents have established views, in a context where mandatory vaccination had generated resentment. Diaz et al. (2025) provide complementary evidence: trust-based messaging from proximate health authorities was among the most effective approaches for HPV uptake in Colombia, consistent with the idea that proximity and existing trust, not distant prestige, drove compliance. Endorsement effectiveness thus depends critically on timing: authority endorsements may work during crises when attitudes are still forming, but backfire for established behaviors in post-coercion contexts – though this conclusion rests on a comparison across studies and requires direct replication.

### 5.2 Pragmatic hesitancy, muslim insulation, and the trust paradox

The endorsement strategy rested on two assumptions: that prestigious messengers can shift health behavior, and that the barriers they address – deference to authority, religious permissibility – are the barriers that actually drive hesitancy. Our qualitative findings (Section 4.2) show the second assumption fails. Hesitancy is pragmatic: efficacy doubts, safety fears, and quality concerns dominate, consistent with Kassabekova et al. (2025)’s COM-B framework analysis of immunization barriers in Kazakhstan. Endorsements that ask respondents to defer to authority rather than providing concrete safety information fail to engage with the actual objections. The Grand Mufti declaring the vaccine “safe and halal” was irrelevant because religion was not the problem — only two of 829 respondents cited religious objections.

Muslim and non-Muslim respondents reacted very differently to the endorsers: non-Muslims’ vaccination intent dropped by 11–12 percentage points across all three endorsers, while Muslims’ intent barely moved. When we split the sample by ethnicity instead of by religion (Supplementary Table 9), the same divide shows up more sharply: ethnic Russian respondents dropped by 15–19 percentage points, while ethnic Kazakh and Other respondents dropped by only 1–3 points. Because 96% of Muslim parents in our sample are ethnic Kazakh and 72% of non-Muslim parents are ethnic Russian, the two cuts are nearly the same cut, and we cannot statistically separate religion from ethnicity. The ethnic gap is the larger of the two, which leads us to read the primary divide as ethnic rather than religious: something about ethnic Russian respondents—not about non-Muslim respondents per se—makes them substantially more negative toward state-linked endorsers.

On top of this broader pattern, the ethnicity analysis shows one messenger-specific effect. Among ethnic Russian respondents, the Grand Mufti pulled vaccination intent down significantly more than the Chief Sanitary Doctor did (Mufti × Ethnic Russian: β=−0.180, p<0.05; Chief Sanitary Doctor × Ethnic Russian: β=−0.119, not significant). This is the pattern a religious-outgroup mechanism would predict: a Muslim cleric is a stronger out-group symbol to ethnic Russian respondents (who are predominantly Orthodox or non-religious) than a secular public-health official is. Our data are therefore consistent with two channels operating at once: a larger ethnic component driving the overall backlash against state-linked messengers, and a smaller religious-outgroup component that is specific to the Mufti among ethnic Russian respondents.^1^

This reading fits with the other evidence in the paper. The absence of Muslim-status moderation on norm messaging (H2c null) is consistent with a religious dimension that matters for a religiously coded messenger but not for aggregate norm information. Hicken et al. (2024)’s null religiosity moderation in Kazakhstan was estimated without contrasting a religious messenger against a secular one, so their finding and ours are compatible: religiosity per se may not predict vaccine attitudes, while a Muslim messenger can still produce a differential response among ethnic Russians.

Government trust clearly structures baseline vaccination attitudes — the qualitative data show a 35-percentage-point gap (75% vs. 40%) between high- and low-trust respondents, consistent with cross-national evidence that trust robustly predicts vaccine willingness (Jennings et al., 2023, Nicholls et al., 2024). Because institutional trust operates as a distinct construct from interpersonal trust with independent effects on vaccine hesitancy (Krastev et al., 2023), the trust deficit driving hesitancy in Kazakhstan likely operates at the level of institutions rather than individual social relationships.

Yet the experimental finding that trust did not buffer the endorsement backfire (H1c) presents a paradox: if trust is the key mechanism, why did high-trust respondents also decline? One explanation is that reactance triggered by autonomy threats operates independently of generalized institutional trust — even respondents who trust the government may resist when specific messages are perceived as directive. Trust predicts baseline attitudes but does not immunize against reactance.

### 5.3 Social norms: Publication bias and the urban–rural paradox

Our null norm effects join growing evidence that published social norms effects are inflated. Papakonstantinou et al. (2025), synthesizing 89 RCTs (n=85,759), found that the uncorrected pooled effect (d=0.14) shrinks to essentially zero after bias correction (d=0.01), with very strong evidence of publication bias (${\text{BF}}_{\text{pb}}=259.54$). Our pre-registered design may provide an unbiased estimate of what social norms messaging actually achieves in practice. However, context matters: Moehring et al. (2023) found that normative information increased COVID-19 vaccine acceptance across 23 countries, suggesting norms may work for novel behaviors where attitudes are still forming but not for routine vaccination where opinions are established. Post-mandate crowding out of voluntary compliance (Schmelz & Bowles, 2021) may further explain the null: coercive policies can weaken the social norms that would otherwise support vaccination.

We predicted, following Rabb et al. (2022), that norm influence would be strongest when reference groups are proximal and concrete — implying rural residents should respond most. The opposite occurred. One plausible reading is that norm verifiability is doing the work: in smaller communities, parents may be in a position to judge a claim like “4 out of 5 residents in your area” against what they see around them, and where the claim does not match, it is likely to be dismissed. In urban settings, direct observation of other parents’ vaccination behavior is more limited, so the same figure is less easily contradicted by personal experience and may be more readily accepted at face value. If so, Rabb et al.’s framework may need qualification: proximity strengthens norm influence only when stated norms are credible; when respondents can check the claim against their own experience, proximity may work against compliance rather than for it. Vriens et al. (2023) found that vaccine-hesitant individuals systematically underestimate community vaccination rates, which is at least consistent with this interpretation. We offer this as a plausible account of the reversal rather than a tested mechanism; our design was not set up to adjudicate between verifiability and alternative explanations such as traditionalism or community-level differences in prior exposure to public health campaigns.

The absence of messenger–norm interactions indicates that prestige bias and conformist bias operate through separate psychological channels. The not-yet-vaccinated subsample (N=529, baseline ≈39%) confirmed zero treatment effects, ruling out ceiling effects as an alternative explanation.

### 5.4 Limitations

Several limitations affect interpretation of our findings. The most important is the messenger–message confound. Our treatments combined specific messengers with message-specific framing language: the Grand Mufti declared the vaccine “safe and halal”, the President “safe and patriotic”, and the Chief Sanitary Doctor “safe and effective”. While this design reflects how these authorities would plausibly communicate in practice, we cannot determine whether the backfire effects stemmed from the authority’s identity, the specific language used, or some combination. The similar magnitude of backfire across all three messengers provides suggestive but not conclusive evidence that messenger identity rather than message content drove the results. Future research should vary messenger identity and message content independently to disentangle these effects.

Second, we measured behavioral intentions rather than actual vaccination behavior. The well-documented intention-behavior gap means our findings indicate potential rather than confirmed effects on real-world vaccination rates.

Third, the qualitative findings are shaped by selection: respondents who answered the optional open-ended question (27% of participants) were more likely to be Russian-speaking, Orthodox Christian, and government-skeptical. Given social stigma attached to vaccine refusal, respondents may have been reluctant to disclose views they perceived as unacceptable. The near-absence of conspiracy theories and religious objections may reflect social desirability rather than true prevalence; the qualitative data tell us what respondents were *willing* to report, which may differ from what actually drives their decisions.

Fourth, our findings pertain specifically to MMR vaccination in Kazakhstan in March 2025 and may not generalize to other vaccines, time periods, or national contexts. We did not conduct gender-disaggregated subgroup analyses; future research should examine whether backfire effects differ by gender.

Finally, two alternative explanations deserve consideration: demand effects (respondents may have inferred the study’s purpose and signaled independence from authority) and treatment intensity (brief text vignettes represent a minimal dose compared to real-world campaigns involving repeated, multi-channel exposure).

### 5.5 Practical implications

The central implication is negative: authority-based endorsements failed and decreased vaccination intent. Practitioners should not assume that identifying prestigious messengers will be effective in settings where institutional trust is low. The declining vaccine confidence documented in post-Soviet countries (de Figueiredo et al., 2020) suggests that authority-based messaging may face similar obstacles throughout the region. Trust-building is a prerequisite, not an outcome of messaging. Cross-sectional evidence confirms that institutional trust independently predicts vaccine skepticism (Roozenbeek et al., 2025), while longitudinal evidence suggests that the attitudinal dispositions underlying vaccine hesitancy – including conspiracy mentality – are stable traits rather than malleable responses to short-term communication (Lamot et al., 2026). Where these trust deficits are entrenched, messaging cannot substitute for credibility.

The language-switching finding – half of Kazakh-speaking respondents writing in Russian – suggests that vaccination discourse occurs primarily in Russian-language media and healthcare settings. Kazakh-dominant speakers may have systematically less access to vaccination information, representing both a gap in current communication and a concrete intervention opportunity.

Because hesitancy stems from pragmatic concerns about efficacy, safety, and quality, messaging that directly addresses these concerns – providing transparent information about vaccine testing, manufacturing standards, and safety monitoring – may be more effective than authority-based appeals. The contingent trust group identified in the qualitative data (9% of respondents) represents persuadable individuals whose decisions depend on transparent evidence rather than authority endorsements.

Healthcare providers who interact directly with patients may be more effective messengers than distant public figures. Attwell et al. (2024) found that trusted community providers and peer outreach were effective strategies for reaching marginalized populations in Australia — groups whose distrust of government institutions parallels the low institutional trust we observe in Kazakhstan. Claessens et al. (2025) report that healthcare providers in Kazakhstan themselves express need for training on vaccination communication, suggesting that provider-level interventions could address both supply-side and demand-side barriers.

Religious framing should be used cautiously in religiously diverse populations. While Muslims were not negatively affected by any endorsement, non-Muslims showed substantial backfire effects. Secular framing grounded in scientific evidence may achieve broader reach.

Social norm messaging may hold promise in urban settings where stated norms are credible because they cannot be directly verified. National campaigns employing identical norm-based messages may be suboptimal; regional tailoring using accurate, locally specific statistics could improve effectiveness.

## 6 Conclusion

Prestige bias and social norms messaging are among the most widely recommended vaccination communication strategies, yet they have been validated almost exclusively in WEIRD, high-trust settings. We tested both in Kazakhstan, a post-Soviet setting where trust in public authorities has been deeply eroded, and found they failed or backfired: all three authority endorsements reduced vaccination intent by 6–7 percentage points, while norm messaging showed null effects. These findings identify boundary conditions for prestige bias theory that prior research, conducted predominantly in high-trust settings, had not fully specified: when trust assumptions are violated, explicit endorsements trigger resistance rather than deference.

The contrast with Hicken et al. (2024)’s positive pandemic-era findings in the same country crystallizes the key insight. Authority endorsements may work during active crises characterized by uncertainty and information-seeking, but backfire for routine vaccines in post-coercion contexts where opinions have formed and resentment has been primed. The effectiveness of prestige-based messaging is not a stable property of endorsers or populations but depends on the trust environment in which communication occurs.

The deeper implication is that the trust deficit is not merely a moderating condition but a structuring one. Where institutions have lost credibility, leveraging institutional authority is not just ineffective but counterproductive — the strategy amplifies the very distrust it needs to overcome. Restoring the conditions under which prestige-based messaging becomes viable requires treating trust-building as a prerequisite to communication, not as its expected outcome.

## CRediT authorship contribution statement

**David Karpa:** Writing – review & editing, Writing – original draft, Project administration, Methodology, Data curation, Formal analysis, Conceptualization. **Dinara Pisareva:** Writing – review & editing, Writing – original draft, Project administration, Methodology, Investigation, Formal analysis, Data curation, Conceptualization. **Bermond Scoggins:** Writing – review & editing, Writing – original draft, Methodology, Software, Formal analysis, Data curation. **Nikita Durnev:** Writing – review & editing, Investigation. **Michael Rochlitz:** Writing – review & editing, Supervision, Project administration, Funding acquisition.

## Ethics statement

This study was approved by the Nazarbayev University Institutional Research Ethics Committee on September 20, 2024 (IREC #937/ 18092024) and by the Central University Research Ethics Committee (CUREC) of the University of Oxford (#662978). All participants provided informed consent. Participation was voluntary; respondents could withdraw at any time. Survey responses were collected anonymously; no personally identifiable information was retained.

## Declaration of Generative AI and AI-Assisted Technologies in the Manuscript Preparation Process

During the preparation of this work, the authors used Claude Code to assist with copyediting, statistical code review, and writing analytical scripts in R. The authors reviewed and edited all AI-assisted output and take full responsibility for the content of the publication.

## Funding

This research was supported by the 10.13039/501100000769University of Oxford, United Kingdom . The funder had no role in study design, data collection, analysis, interpretation, or the decision to submit for publication.

## Declaration of competing interest

The authors declare that they have no known competing financial interests or personal relationships that could have appeared to influence the work reported in this paper.

## Data Availability

The data and replication code are openly available via Discuss Data at https://doi.org/10.48320/6C9610DC-8456-44B6-9165-3F0A562B7E40.

## Appendix A Supplementary data

MMC S1 Supplementary tables and figures: robustness checks, subgroup analyses, and qualitative coding details. MMC S1

## Footnotes

1 The parochialism literature (Pisor & Ross, 2024) offers a related framing in which ingroup favoritism and outgroup hostility are analytically distinct and group boundaries are fluid; while that framework is developed for interpersonal and intergroup altruism rather than citizen–state relations, it is consistent with the idea that group boundaries shape responses to messengers.
