# Supplementary material for: Authority endorsements backfire and social norms fail to increase vaccination intent in post-COVID Kazakhstan
Source: SSM Popul Health. 2026 Jun 20;35:101935. doi: 10.1016/j.ssmph.2026.101935 (PMC13321262; doi:10.1016/j.ssmph.2026.101935)
Supplement: MMC S1 — Supplementary tables and figures: robustness checks, subgroup analyses, and qualitative coding details. [file mmc1.pdf]

Appendix

A Model Specifications

All models are estimated on the parent subsample ($n = 1{,}420$) using linear probability models with heteroskedasticity-robust (HC2) standard errors and survey weights. The outcome $Y_i \in \{0, 1\}$ is vaccination intent.

Main effects. We estimate separate models for each treatment dimension:

$$Y_i = \alpha + \sum_{j=1}^{3} \beta_j \, \text{Endorser}_{ij} + \varepsilon_i \tag{1}$$

$$Y_i = \alpha + \sum_{k=1}^{3} \gamma_k \, \text{Norm}_{ik} + \varepsilon_i \tag{2}$$

where Endorser_{ij} and Norm_{ik} are indicator variables for each treatment condition (Grand Mufti, President, CSD; Religious Norm, National Norm, Local Norm) with the control condition as the reference category.

Interaction model. To test for messenger–norm interactions:

$$Y_i = \alpha + \sum_{j=1}^{3} \beta_j \, \text{Endorser}_{ij} + \sum_{k=1}^{3} \gamma_k \, \text{Norm}_{ik} + \sum_{j=1}^{3} \sum_{k=1}^{3} \delta_{jk} \, \text{Endorser}_{ij} \times \text{Norm}_{ik} + \varepsilon_i \tag{3}$$

Subgroup analyses. For each hypothesized moderator M_i (Muslim status for H1b and H2c, government trust for H1c, major city for H2b), we interact the relevant treatment dimension with the moderator:

$$Y_i = \alpha + \sum_{j=1}^{3} \beta_j \, \text{Treatment}_{ij} + \phi \, M_i + \sum_{j=1}^{3} \lambda_j \, \text{Treatment}_{ij} \times M_i + \varepsilon_i \tag{4}$$

Region fixed effects (robustness). We re-estimate the main effects models adding region indicators:

$$Y_i = \alpha + \sum_{j=1}^{3} \beta_j \, \text{Treatment}_{ij} + \sum_{r=1}^{18} \rho_r \, \text{Region}_{ir} + \varepsilon_i \tag{5}$$

with one region as the reference category ($R = 19$ regions, 18 indicators). Results are reported in Table 14.

B Tables

Table 5: Sample Distribution by Region

	Full Sample		Parent Subsample	
Region	N	%	N	%
Abay Region	42	1.3	15	1.1
Akmola Region	125	4.0	67	4.7
Aktobe Region	124	4.0	55	3.9
Almaty	370	11.8	191	13.5
Almaty Region	166	5.3	71	5.0
Astana	227	7.3	104	7.3
Atyrau Region	94	3.0	37	2.6
East Kazakhstan Region	189	6.0	82	5.8
Karaganda Region	208	6.7	107	7.5
Kostanay Region	121	3.9	63	4.4
Kyzylorda Region	213	6.8	95	6.7
Mangystau Region	91	2.9	53	3.7
North Kazakhstan Region	106	3.4	55	3.9
Other	283	9.1	120	8.5
Pavlodar Region	154	4.9	60	4.2
Shymkent	171	5.5	81	5.7
Turkistan Region	263	8.4	97	6.8
Ulytau Region	20	0.6	5	0.4
Zhambyl Region	157	5.0	62	4.4

Table 6: Sample Distribution by Categorical Variables

		Full Sample		Parent Subsample	
Variable	Category	N	%	N	%
Ethnicity	Kazakh	2028	64.9	944	66.5
	Russian	965	30.9	417	29.4
	Other	131	4.2	59	4.2
Occupation	Private company	898	28.7	488	34.4
	Government organization	354	11.3	227	16.0
	Self-employed	437	14.0	129	9.1
	Business owner	73	2.3	47	3.3
	NGO	59	1.9	40	2.8
	Employed in farming	42	1.3	18	1.3
	Law enforcement/security	32	1.0	16	1.1
	Other	32	1.0	13	0.9
	NA	1197	38.3	442	31.1
Financial Situation	Enough for food/clothing, not durables	1466	46.9	640	45.1
	Can afford everything except expensive	809	25.9	394	27.7
	Enough for food but not clothing	533	17.1	250	17.6
	Not enough money for food	218	7.0	109	7.7
	No financial difficulties	98	3.1	27	1.9

Table 7: Interaction Effects on Vaccine Intent

	Interaction Model
Intercept (Control × Control)	0.790***
	(0.047)
Chief Sanitary Doctor	−0.042
	(0.065)
President Tokayev	−0.110
	(0.072)
Grand Mufti of Kazakhstan	−0.096
	(0.068)
Local Norm	−0.054
	(0.067)
National Norm	−0.131+
	(0.068)
Religious Norm	0.062
	(0.059)
Chief Doctor × Local Norm	−0.052
	(0.097)
President × Local Norm	0.060
	(0.099)
Grand Mufti × Local Norm	0.083
	(0.096)
Chief Doctor × National Norm	0.104
	(0.095)
President × National Norm	0.228*
	(0.099)
Grand Mufti × National Norm	0.108
	(0.098)
Chief Doctor × Religious Norm	−0.143
	(0.090)
President × Religious Norm	−0.117
	(0.092)
Grand Mufti × Religious Norm	−0.066
	(0.091)
Num. Obs.	1420
R^2	0.017
R^2 Adj.	0.007

Notes: + $p < 0.1$, * $p < 0.05$, ** $p < 0.01$, *** $p < 0.001$. Reference category is Control × Control.

Table 8: Heterogeneous Treatment Effects by Muslim Status (Endorser)

	Endorser × Muslim
Intercept (Control × Non-Muslim)	0.753***
	(0.036)
Grand Mufti of Kazakhstan	−0.116*
	(0.054)
President Tokayev	−0.114*
	(0.055)
Chief Sanitary Doctor	−0.122*
	(0.055)
Muslim	0.010
	(0.046)
Grand Mufti × Muslim	0.085
	(0.068)
President × Muslim	0.065
	(0.069)
Chief Doctor × Muslim	0.095
	(0.070)
Num. Obs.	1420

Notes: + $p < 0.1$, * $p < 0.05$, ** $p < 0.01$, *** $p < 0.001$. Reference category is Control × Non-Muslim.

Table 9: Heterogeneous Treatment Effects by Ethnicity (Exploratory)

	Endorser × Ethnic Russian
Intercept (Control × Ethnic Kazakh/Other)	0.761***
	(0.027)
Grand Mufti of Kazakhstan	−0.012
	(0.038)
President Tokayev	−0.030
	(0.039)
Chief Sanitary Doctor	−0.031
	(0.039)
Ethnic Russian	−0.004
	(0.050)
Grand Mufti × Ethnic Russian	−0.180*
	(0.074)
President × Ethnic Russian	−0.144+
	(0.074)
Chief Doctor × Ethnic Russian	−0.119
	(0.076)
Num. Obs.	1420

Notes: + $p < 0.1$, * $p < 0.05$, ** $p < 0.01$, *** $p < 0.001$. Reference category is Control × Ethnic Kazakh/Other. Ethnic Russian = 1 if the respondent self-identified as ethnic Russian, 0 otherwise (Kazakh or Other). This analysis is exploratory: ethnicity was not pre-registered as a subgroup moderator.

Table 10: Heterogeneous Treatment Effects by Government Trust

	Endorser × Government Trust
Intercept (Control × Low Trust)	0.682***
	(0.041)
Grand Mufti of Kazakhstan	−0.035
	(0.060)
President Tokayev	−0.063
	(0.061)
Chief Sanitary Doctor	−0.018
	(0.060)
High Government Trust	0.123*
	(0.049)
Grand Mufti × High Trust	−0.054
	(0.072)
President × High Trust	−0.023
	(0.072)
Chief Doctor × High Trust	−0.075
	(0.072)
Num. Obs.	1420

Notes: + $p < 0.1$, * $p < 0.05$, ** $p < 0.01$, *** $p < 0.001$. Reference category is Control × Low Trust. High Trust = Somewhat trust or Completely trust.

Table 11: Heterogeneous Treatment Effects by Muslim Status (Norm Type)

	Norm Type × Muslim
Intercept (Control × Non-Muslim)	0.662***
	(0.041)
Four out of five Muslims	0.001
	(0.055)
Four out of five citizens	0.015
	(0.058)
Four out of five residents	0.002
	(0.058)
Muslim	0.111*
	(0.050)
Religious Norm × Muslim	−0.032
	(0.069)
National Norm × Muslim	−0.067
	(0.072)
Local Norm × Muslim	−0.057
	(0.072)
Num. Obs.	1420

Notes: + $p < 0.1$, * $p < 0.05$, ** $p < 0.01$, *** $p < 0.001$. Reference category is Control × Non-Muslim.

Table 12: Heterogeneous Treatment Effects by Location Type

	Norm Type × Major City
Intercept (Control × Regional)	0.745***
	(0.027)
Four out of five Muslims	−0.073+
	(0.039)
Four out of five citizens	−0.070+
	(0.040)
Four out of five residents	−0.068+
	(0.040)
Major City	−0.078
	(0.061)
Religious Norm × Major City	0.204**
	(0.078)
National Norm × Major City	0.192*
	(0.080)
Local Norm × Major City	0.146+
	(0.081)
Num. Obs.	1420

Notes: + $p < 0.1$, * $p < 0.05$, ** $p < 0.01$, *** $p < 0.001$. Reference category is Control × Regional. Major City = Almaty, Astana, or Shymkent.

Table 13: Treatment Effects in the Full Sample (Including Non-Parents)

	Endorser Type	Norm Type
Intercept (Control)	0.678***	0.667***
	(0.017)	(0.017)
Grand Mufti of Kazakhstan	−0.027	
	(0.024)	
President Tokayev	−0.040	
	(0.024)	
Chief Sanitary Doctor	0.001	
	(0.024)	
Four out of five Muslims		0.005
		(0.024)
Four out of five citizens		−0.017
		(0.024)
Four out of five residents		−0.008
		(0.024)
Num. Obs.	3124	3124
R^2	0.001	0.000

Notes: + $p < 0.1$, * $p < 0.05$, ** $p < 0.01$, *** $p < 0.001$. Full survey sample including non-parents. Same specification as Table 3. All treatment effects are near zero and insignificant, confirming that the endorsement backfire effect observed in the parent subsample does not generalize to respondents without immediate vaccination decisions.

Table 14: Robustness: Main Treatment Effects with Region Fixed Effects

	Endorser		Norm	
	Baseline	Region FE	Baseline	Region FE
Intercept (Control)	0.760***	0.872***	0.728***	0.824***
	(0.023)	(0.108)	(0.024)	(0.110)
Grand Mufti of Kazakhstan	−0.066*	−0.082*		
	(0.033)	(0.033)		
President Tokayev	−0.074*	−0.086**		
	(0.033)	(0.033)		
Chief Sanitary Doctor of Kazakhstan	−0.063+	−0.080*		
	(0.034)	(0.033)		
Four out of five Muslims			−0.020	−0.013
			(0.034)	(0.034)
Four out of five citizens			−0.023	−0.027
			(0.034)	(0.034)
Four out of five residents			−0.030	−0.035
			(0.035)	(0.035)
Num. Obs.	1420	1420	1420	1420
R^2	0.004	0.049	0.001	0.043
R^2 Adj.	0.002	0.034	−0.002	0.029
Region Fixed Effects	No	Yes	No	Yes

Notes: + $p < 0.1$, * $p < 0.05$, ** $p < 0.01$, *** $p < 0.001$. Region fixed effects (19 regions) included in columns 2 and 4; coefficients not shown. Reference category is Control. HC2 robust standard errors in parentheses. Same specification as Table 3 with the addition of region indicators.

C Supplementary Figures

Figure S1: Predicted probability of vaccination intent across all 16 experimental cells (n=1,420).

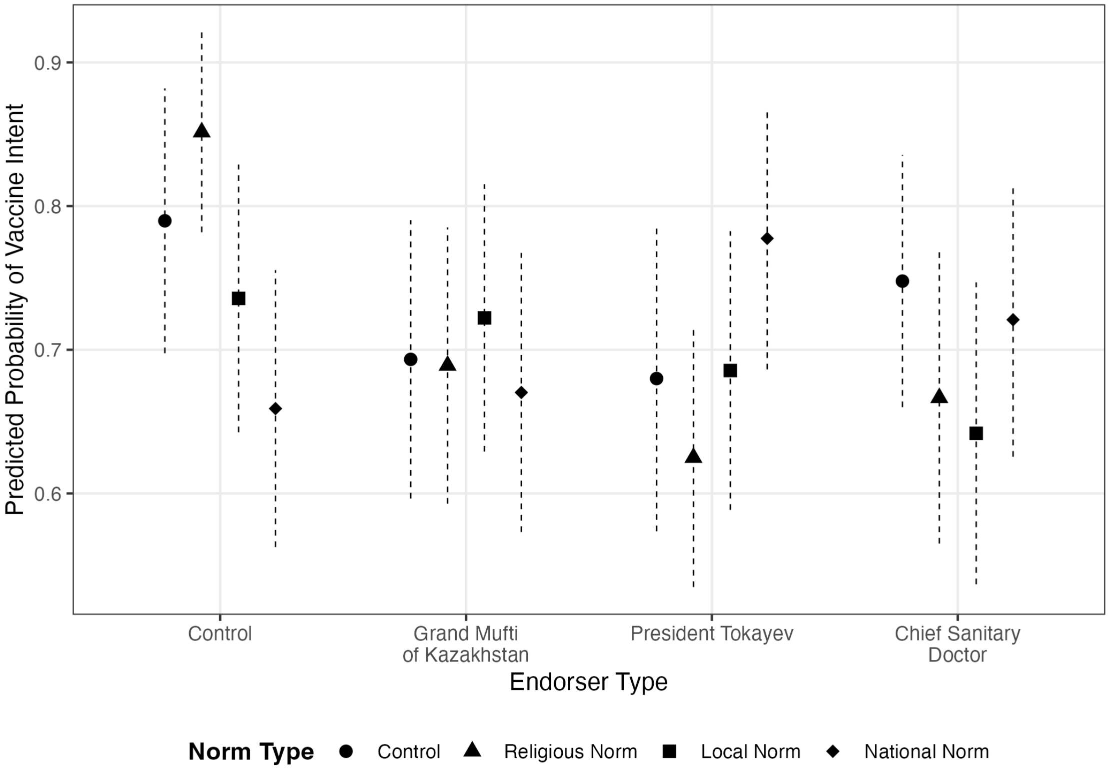

Each point represents a cell-level predicted probability with 95% confidence intervals from a fully interacted linear probability model (4 endorser types × 4 norm types). Different shapes indicate norm type conditions within each endorser condition. The figure shows no systematic interaction pattern: negative endorser effects appear to operate independently of norm treatments, with no evidence that particular messenger–norm combinations produce synergistic or countervailing effects.

Figure S2: Interaction contrasts: norm effects within each endorser condition (n=1,420).

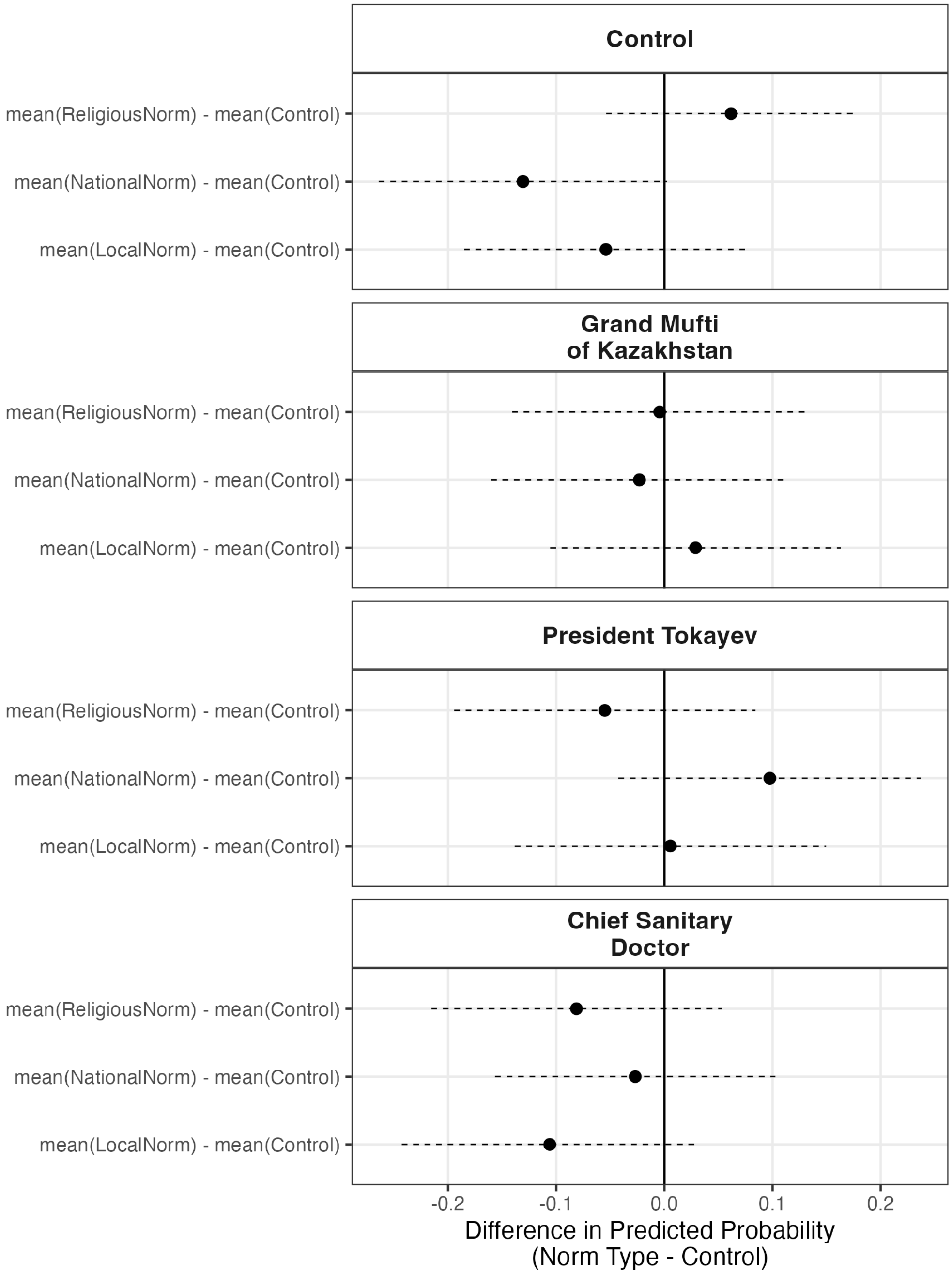

Difference in predicted probability of vaccination intent between each norm condition and the no-norm control, estimated separately within each endorser condition. Horizontal lines at zero indicate no difference from the norm control. Confidence intervals consistently span zero across all endorser–norm combinations, confirming that endorser and norm effects operate independently rather than interactively.

Figure S3: Muslim–non-Muslim contrasts in vaccination intent by endorser condition (n=1,420).

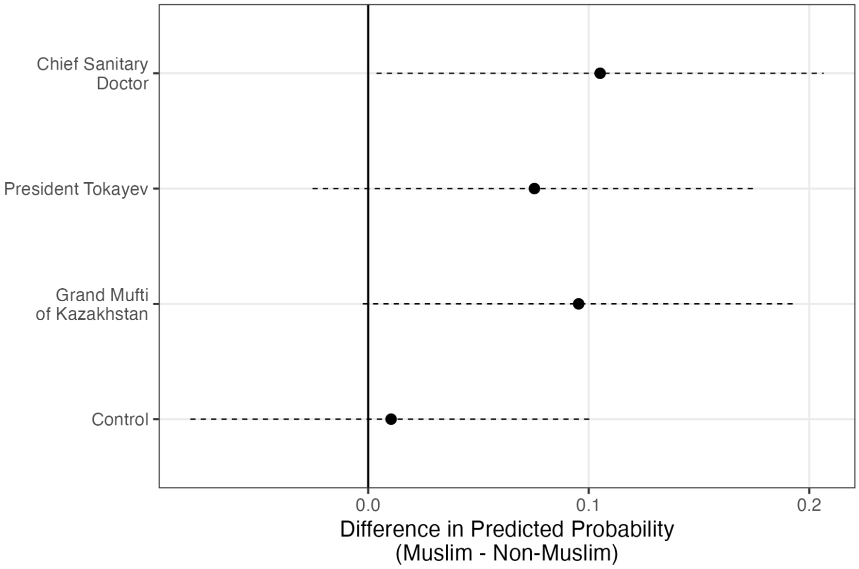

Positive values indicate higher intent among Muslims. In the control condition, Muslim and non-Muslim intent is similar. In all three endorser conditions, Muslims show substantially higher intent than non-Muslims, reflecting the differential backfire effect rather than a positive Muslim response to endorsements.

Figure S4: Heterogeneous endorser effects by ethnicity (n=1,420, exploratory).

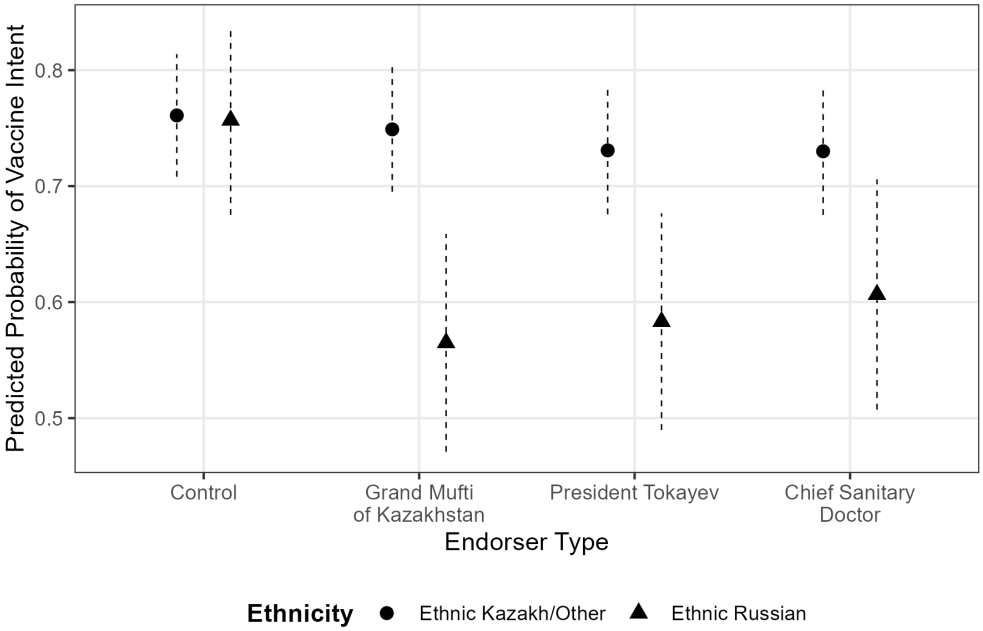

Predicted probabilities of vaccination intent for ethnic Russian respondents (n=417) and all other respondents (ethnic Kazakh or Other, n=1,003) across endorser conditions, with 95% confidence intervals. Ethnic Russians show sharp declines under all three endorsers (Grand Mufti −19.2 pp; President −17.4 pp; Chief Sanitary Doctor −15.0 pp) relative to the control. Ethnic Kazakh/Other respondents show small, non-significant declines (−1 to −3 pp). Ethnicity was not pre-registered as a subgroup moderator; this analysis is exploratory.

Figure S5: Ethnic Russian vs. Ethnic Kazakh/Other contrasts in vaccination intent by endorser condition (n=1,420, exploratory).

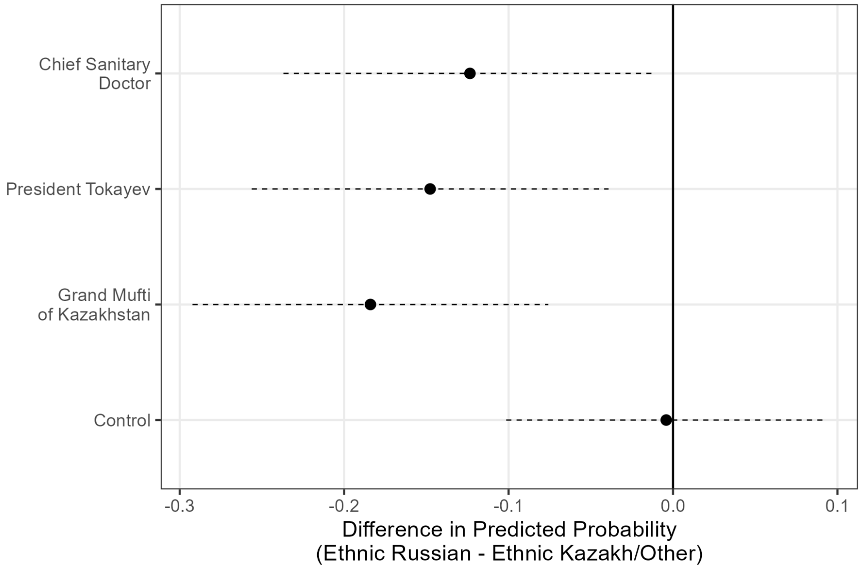

Negative values indicate lower intent among ethnic Russians relative to ethnic Kazakh/Other respondents in the same endorser condition. The ethnic gap is small in the control condition and widens sharply under all three endorsers, with the Grand Mufti producing the largest gap.

Figure S6: Heterogeneous endorser effects by government trust (n=1,420).

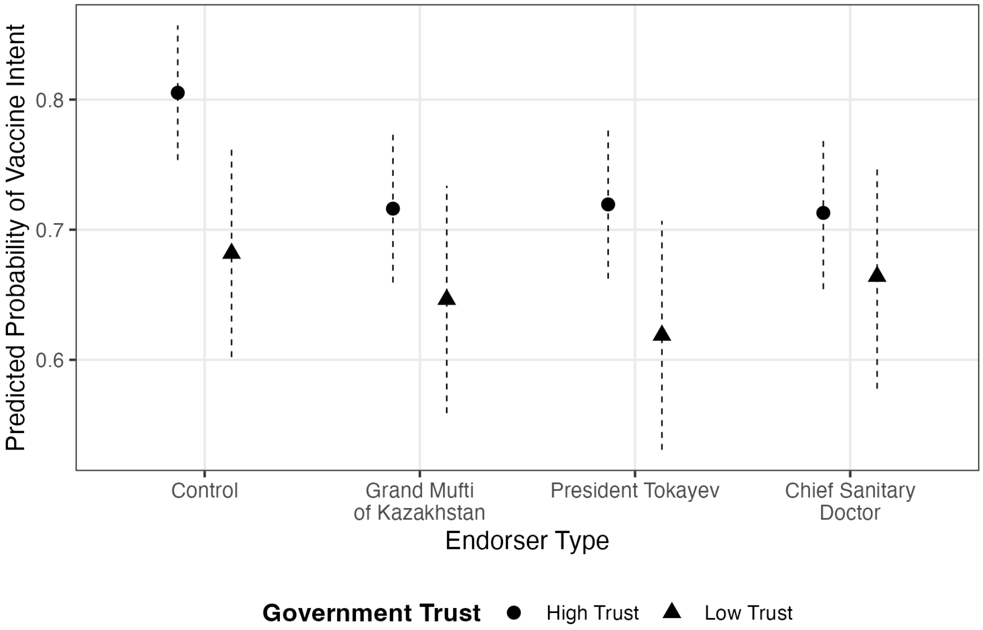

Predicted probabilities of vaccination intent for high-trust ("Somewhat trust" or "Completely trust") and low-trust ("Do not trust much" or "Do not trust at all") respondents across endorser conditions, with 95% confidence intervals. High-trust respondents exhibit higher baseline intent in the control condition (≈81% vs. ≈68%), but both groups experience declines under all three endorser treatments. The trust gap narrows in treatment conditions, providing no evidence that government trust enhances receptivity to political endorsements.

Figure S7: High-trust minus low-trust contrasts in vaccination intent by endorser condition (n=1,420).

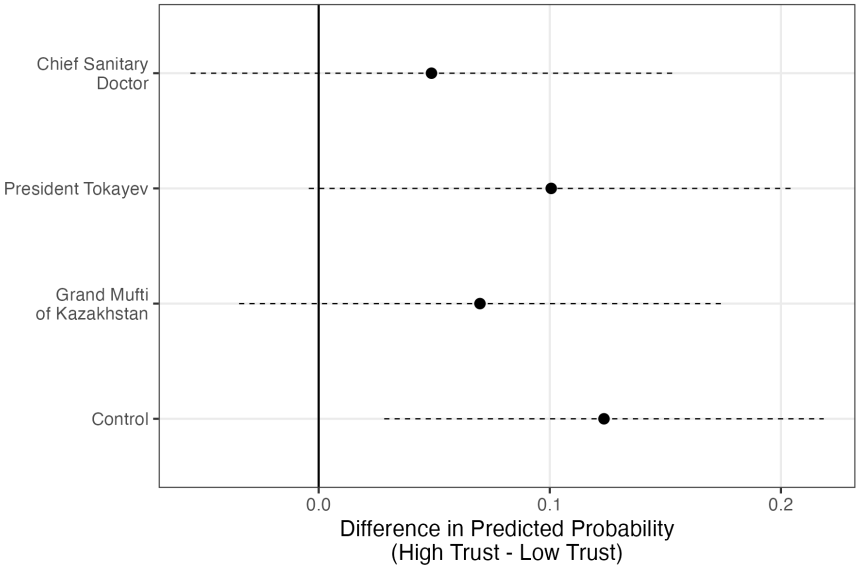

Positive values indicate higher intent among high-trust respondents. The trust gap is largest in the control condition (≈12 percentage points) and narrows in all treatment conditions, particularly for the Chief Sanitary Doctor (≈5 points). This narrowing reflects parallel declines rather than differential persuasion among high-trust respondents.

Figure S8: Major city minus regional contrasts in vaccination intent by norm condition (n=1,420).

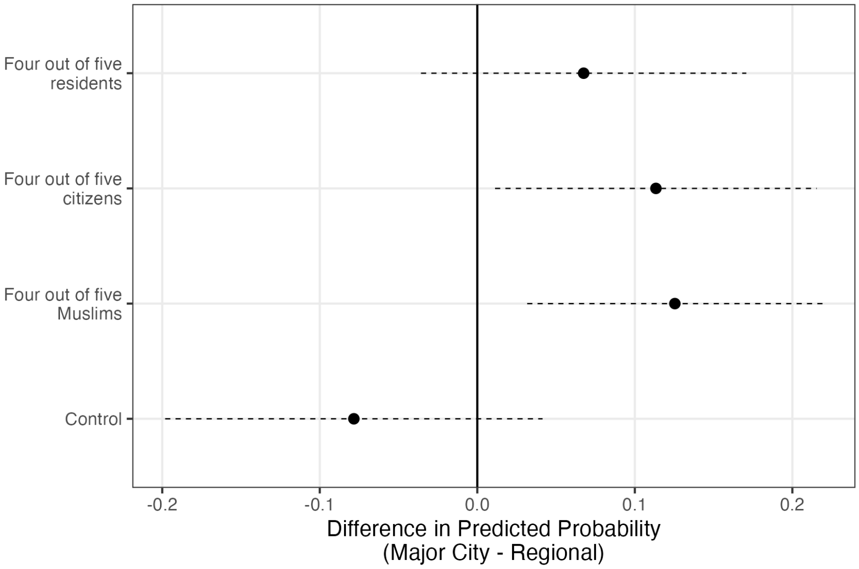

Positive values indicate higher intent among major city respondents. In the control condition, major city residents show lower intent (≈−8 points), but this reverses under all norm treatments (≈+6 to +12 points). Wide confidence intervals reflect the exploratory nature of these interaction analyses.

Figure S9: Heterogeneous norm effects by Muslim status (n=1,420).

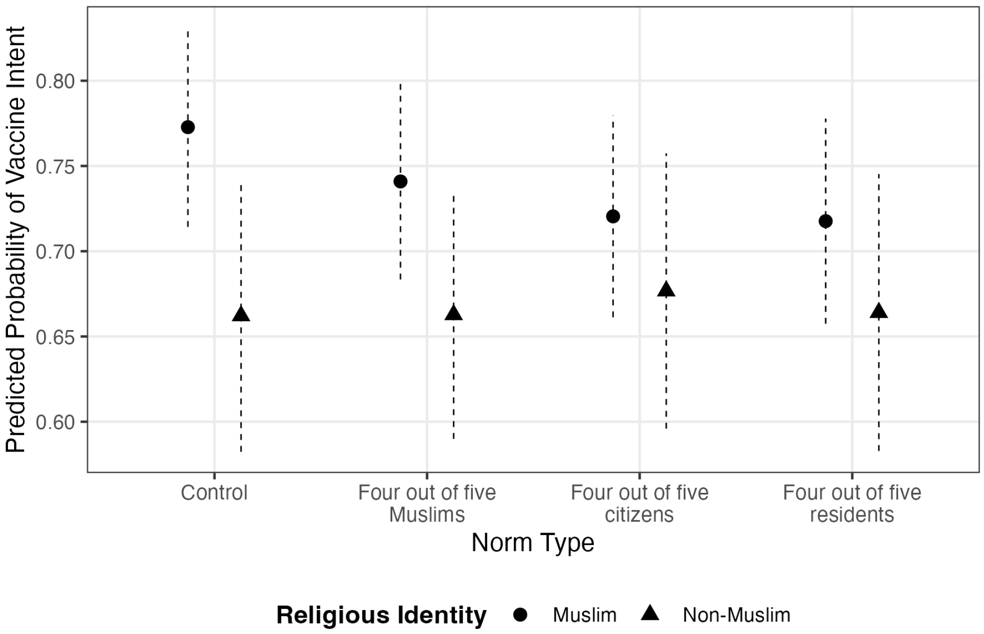

Predicted probabilities of vaccination intent for Muslim and non-Muslim respondents across norm conditions, with 95% confidence intervals. Unlike the strong moderation observed for endorser effects, Muslim status does not substantially moderate responses to social norm messaging. Both groups show similar, largely null responses to all three norm treatments.

Figure S10: Muslim–non-Muslim contrasts in vaccination intent by norm condition (n=1,420).

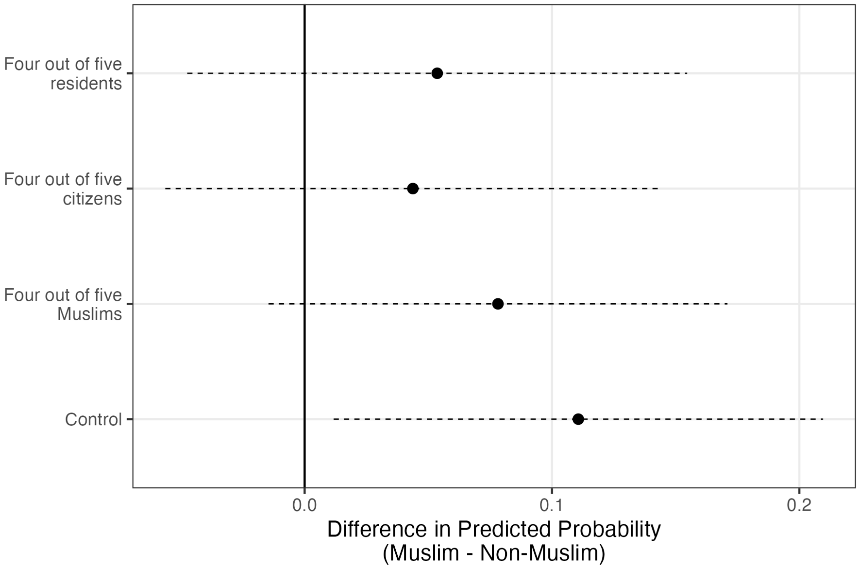

Positive values indicate higher intent among Muslims. The Muslim advantage is relatively consistent across norm conditions, with no evidence that religious community norms differentially affect Muslim respondents, contrary to H2c.

Figure S11: Language flow from survey language choice to open-ended response language.

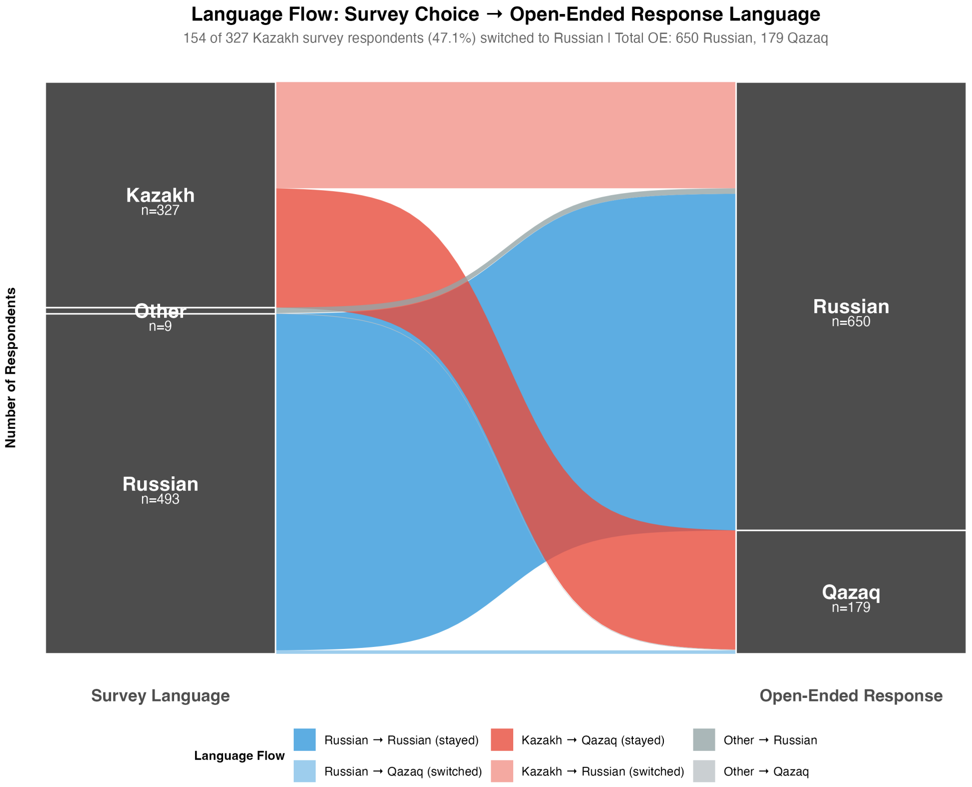

Figure S12: Co-occurrence matrix showing how often qualitative codes appear together within the same response. Darker cells indicate more frequent co-occurrence.

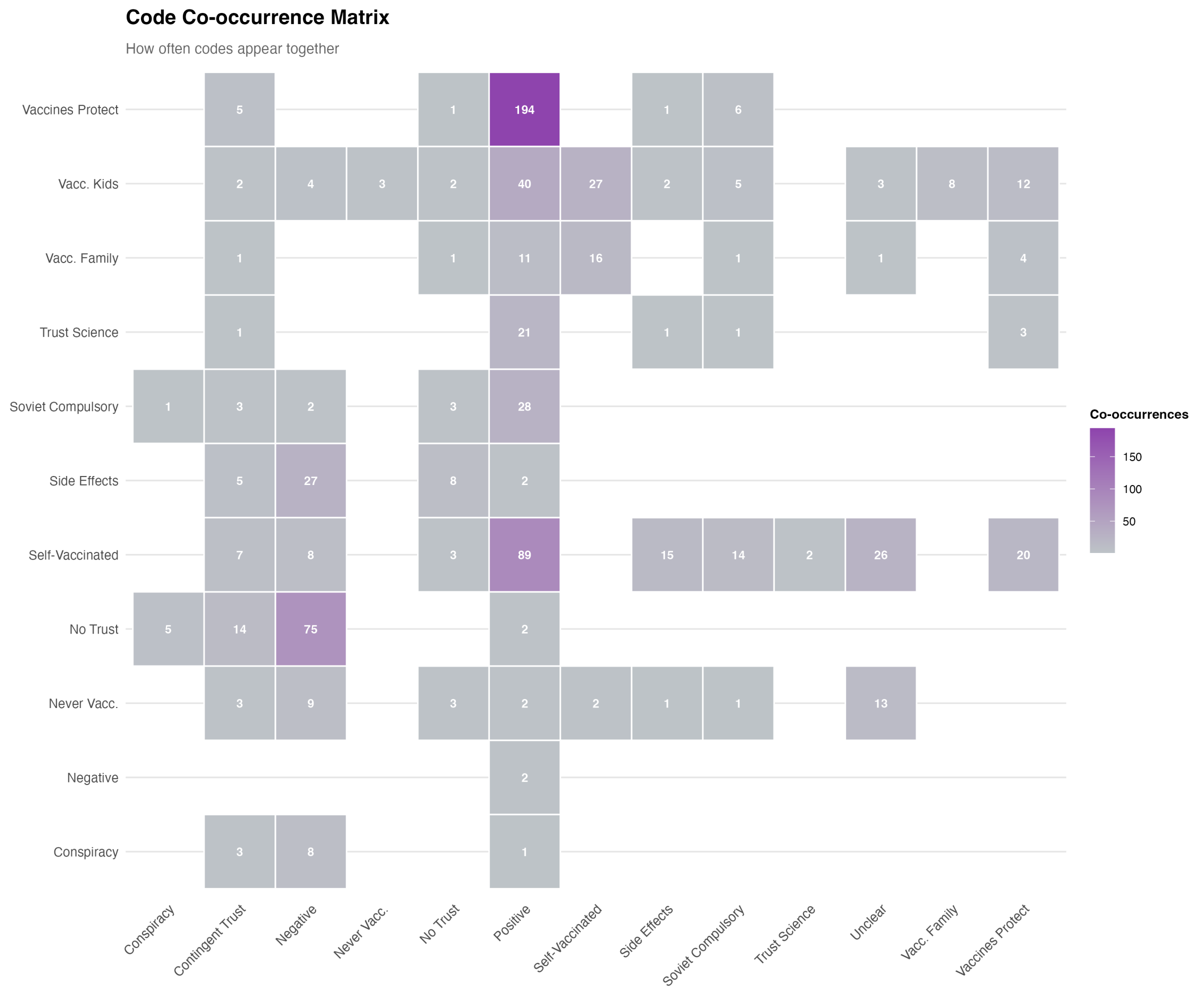

D Survey Instrument

The English-language version of the survey instrument is reproduced below. The Russian and Kazakh source versions were administered to respondents and are available from the authors on request. Variable names used in the dataset are shown in monospace after each question.

Demographics and Background

Please indicate your age. `[age]`
Empty field for a value between 18 and 100. If the value is <18, the survey ends. If the value is >100, an error message appears. Only whole numbers are allowed. A quota check will be performed based on the respondent's age.

In which region do you primarily reside? `[region]`
A quota check will be performed based on the respondent's region.

- Astana; Almaty; Shymkent
- Abay, Akmola, Aktobe, Almaty, Atyrau, East Kazakhstan, Zhambyl, Zhetisu, West Kazakhstan, Karaganda, Kostanay, Kyzylorda, Mangystau, Pavlodar, North Kazakhstan, Turkistan, or Ulytau Region
- Other (Please specify) — Survey ends with a message: "Thank you for your willingness to participate."

Please indicate your gender. `[gender]`
A quota check will be performed based on the respondent's gender.

- Male
- Female

Which of the following best describes your employment status in the past month? `[employment_status]`

- Employed full-time
- Employed part-time
- On parental leave
- Homemaker
- Unemployed and actively looking for work
- Unemployed and not looking for work
- Student
- Retired
- Not working due to health reasons/disability

- Other (Please specify)

Where have you primarily worked in the past month? `[occupation]`
Only asked of respondents who indicated full-time or part-time employment.

- Private company
- Law enforcement / security agencies
- Government organization
- Self-employed (e.g., taxi/bus driver, tailor, baker, construction worker, general laborer, etc.)
- Business owner
- Employed in a farming enterprise
- Non-governmental organization
- Other (Please specify)

How many children under 18 years old do you have? `[children]`

- No children under 18
- 1; 2; 3; 4; 5 or more

Have you (or do you plan to) vaccinate your children against measles, mumps, and rubella? `[vacc_status]`

- Yes
- No
- Prefer not to answer

How would you describe your family's financial situation? `[financial_situation]`

- We do not have enough money for food
- We have enough money for food, but not for clothing
- We have enough money for food and clothing, but purchasing durable goods (e.g., a TV or refrigerator) is difficult
- We have enough money for everything except very expensive purchases like a car or an apartment
- We do not experience financial difficulties and can make any necessary purchase

What is your ethnicity? `[ethnicity]`

- Kazakh
- Russian
- Other (please specify)

Which religious affiliation do you identify with? `[confession]`

- Islam
- Orthodox Christianity
- Catholicism or Protestantism
- Judaism
- Buddhism
- Hinduism
- I do not consider myself religious
- Other (please specify)

How often do you engage in religious or prayer practices? `[religiosity]`
Skipped if the respondent indicated no religious affiliation in the previous question.

- Every day
- At least once a week
- At least once a month
- At least once a year
- Never

What is your highest level of education? `[education]`

- Incomplete secondary education (school)
- Completed secondary education (school)
- Incomplete vocational education (technical school, lyceum, college, etc.)
- Completed vocational education (technical school, lyceum, college, etc.)
- Incomplete higher education
- Completed higher education
- No formal education

What is your citizenship? `[citizenship]`

- Kazakhstan; Russia; Kyrgyzstan; Uzbekistan; Tajikistan; Turkmenistan
- Other (please specify)

What type of locality do you live in? `[residency]`
If the respondent indicated Astana, Shymkent, or Almaty for their region, this question is skipped and "City of national significance" is automatically assigned.

- City, regional center
- City, but NOT a regional center
- Urban-type settlement
- Village or rural area

What is the name of your locality? `[locality]`
Open-ended text box, with routing rules that skip and auto-assign the locality name based on prior region and locality-type responses.

- Please specify (open-ended text box)
- Prefer not to answer

Which languages do you speak fluently? `[language_kazakh, language_russian, language_english, language_turkish, language_other]`
Multiple choice.

- Kazakh; Russian; English; Turkish
- Other (please specify)

What language do you use most frequently at home? `[language_home]`

- Kazakh; Russian; English; Turkish
- Other (please specify)

Trust and International Opinions

To what extent do you think most people can be trusted? `[social_trust]`

- Can be fully trusted
- Can be trusted to some extent
- Should not be trusted too much
- Cannot be trusted at all

How much do you trust the government of Kazakhstan? `[government_trust]`

- Completely trust
- Somewhat trust
- Do not trust much
- Do not trust at all

What is your overall opinion of Russia? `[opinion_russia]`

- Very positive; Positive; Neutral; Negative; Very negative

What is your overall opinion of China? `[opinion_china]`

- Very positive; Positive; Neutral; Negative; Very negative

What is your overall opinion of the United States of America (USA)? `[opinion_us]`

- Very positive; Positive; Neutral; Negative; Very negative

Attention Check

How old are you? `[age_check]`
Quality control: the age group should match the initial age response, with a ±1 year discrepancy allowed. After two failed attempts the respondent is removed for failing quality criteria.

- Under 18; 18–25; 26–35; 36–45; 46–55; 56–65; Over 65

Vaccination Experiment

All respondents answer except those who indicated no children under 18.

Respondents were randomly assigned to one cell of a 4 × 4 factorial design crossing a messenger-endorsement treatment with a descriptive-norm treatment. The messenger treatments (rows) and norm treatments (columns) are shown below.

Messenger-endorsement treatment arms:

- *Grand Mufti of Kazakhstan.* "To combat measles and its severe consequences for children, the Grand Mufti of Kazakhstan, Nauryzbai Otpenov, has approved the measles, mumps, and rubella (MMR) vaccine for all citizens of Kazakhstan. The Grand Mufti made a public statement affirming that the vaccine is safe and *halal*."
- *President.* "To combat measles and its severe consequences for children, President Kassym-Jomart Tokayev has approved the measles, mumps, and rubella (MMR) vaccine for all citizens of Kazakhstan. The President made a public statement affirming that the vaccine is safe and patriotic."

- *Chief Sanitary Doctor.* "To combat measles and its severe consequences for children, the Chief Sanitary Doctor of Kazakhstan, Sarkhat Beisenova, has approved the measles, mumps, and rubella (MMR) vaccine for all citizens of Kazakhstan. The Chief Sanitary Doctor made a public statement affirming that the vaccine is safe and effective."
- *Control.* No messenger message.

Descriptive-norm treatment arms:

- *Religious in-group norm.* "Four out of five Muslims in Kazakhstan have already vaccinated their children with the measles, mumps, and rubella (MMR) vaccine."
- *National norm.* "Four out of five citizens of Kazakhstan have already vaccinated their children with the measles, mumps, and rubella (MMR) vaccine."
- *Local norm.* "Four out of five residents in your district have already vaccinated their children with the measles, mumps, and rubella (MMR) vaccine."
- *Control.* No norm message.

Do you plan to vaccinate your children against measles, mumps, and rubella, or have they already been vaccinated? Hypothetically, if you did not have children, would you vaccinate them? `[vacc_intent]`

- Yes
- No

Can you describe your vaccination experience and the reasons why you generally support or do not support vaccination for yourself and/or your family members? `[vacc_experience]`

- Open-ended text
- Prefer not to answer
